# Advances in organocatalyzed synthesis of organic compounds

**DOI:** 10.1039/d4ra03046j

**Published:** 2024-06-25

**Authors:** Ayesha Zafar, Muhammad Adnan Iqbal, Ghazala Iram, Umar Sohail Shoukat, Faisal Jamil, Muhammad Saleem, Muhammad Yousif, Zain ul Abidin, Mohammad Asad

**Affiliations:** a Department of Chemistry, University of Agriculture Faisalabad Faisalabad-38000 Pakistan adnan.iqbal@uaf.edu.pk; b Organometallic and Coordination Chemistry Laboratory, Department of Chemistry, University of Agriculture Faisalabad Faisalabad-38000 Pakistan; c Department of Basic and Applied Chemistry, Faculty of Sciences and Technology, University of Central Punjab Lahore Pakistan; d Center of Excellence for Advanced Materials Research (CEAMR), King Abdulaziz University P.O. Box 80203 Jeddah 21589 Saudi Arabia

## Abstract

The recent advancements in utilizing organocatalysts for the synthesis of organic compounds have been described in this review by focusing on their simplicity, effectiveness, reproducibility, and high selectivity which lead to excellent product yields. The organocatalytic methods for various derivatives, such as indoles, pyrazolones, anthrone-functionalized benzylic amines, maleimide, polyester, phthalimides, dihydropyrimidin, heteroaryls, *N*-aryl benzimidazoles, stilbenoids, quinazolines, quinolines, and oxazolidinones have been specifically focused. The review provides more understanding by delving into potential reaction mechanisms. We anticipate that this collection of data and findings on successful synthesis of diverse compound derivatives will serve as valuable resources and stimulating current and future research efforts in organocatalysis and industrial chemistry.

## Abstract 2

Recent advancements in utilizing organocatalysts emphasize their simplicity, effectiveness, reproducibility, and high selectivity, resulting in excellent yields of heterocyclic organic compounds.

## Graphical abstract

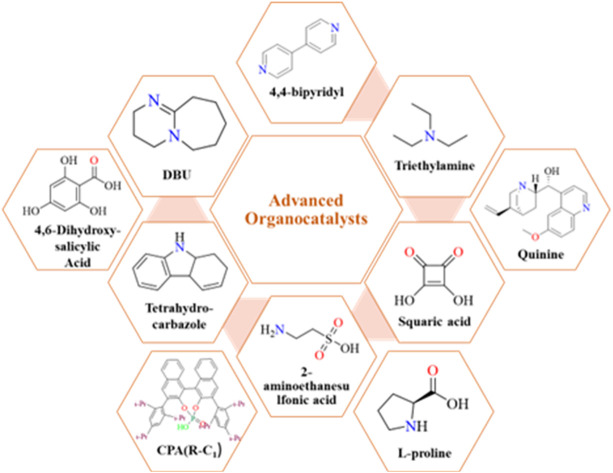

## 1 Introduction

Numerous techniques and effective catalysts have been developed to create synthetic organic compounds, which are in higher demand due to their low cost, simplicity of use, increased color stability, and resistance to light and pH changes. In order for modern civilization to successfully advance economically, it is essential that synthetic chemicals be produced in large quantities at low cost.^1^ With the use of catalysts, it is theoretically possible to quickly and selectively synthesize desired chemical molecules without using additional energy or the catalyst itself.^1,2^ Catalysis is the current foundation for the majority of modern chemical processes. Growing environmental consciousness and increased pressure on the chemical sector to reduce waste generation both support the gradual replacement of conventional stoichiometric methods with cleaner catalytic substitutes. In contrast to the bulk chemicals and oil refining industries, which typically produce about 0.1 and 1–5 kg of byproducts per kilogram of product, respectively, the quantity of waste produced per kilogram of product, or E factor, is particularly high in the production of fine chemicals (up to 100 kg). This is partly because fine chemistry uses multi-step synthesis, but it's also because stoichiometric rather than catalytic techniques are used more frequently.^3^

Recently, it has become more and more important to use tiny organic compounds as catalysts. Compared to the organometallic catalysts typically predominate in asymmetric synthesis, it has several advantages, such as decreased toxicity, greater stability, reduced activation energy, and friendly to the environment. Because of their low contamination and lack of metal traces in products, they facilitate the best reaction conditions, prevent metallic waste, and are perfect for the pharmaceutical industry.^4–7^ This article aims to quickly display certain classic and contemporary publications explaining, how these catalysts play crucial role in synthesis of organic components. In literature, recent work reported on organocatalysis includes organocatalysis of pyrrole and amino acids,^8,9^ dehydroabietane-type bifunctional organocatalysis,^10^ degradation of polluting dyes,^11–13^ asymmetric photocatalysis,^14^ proline derivatives catalyzed reactions in aqueous media,^15–17^ ionic liquid as organocatalysts,^18,19^ guanidinium catalyzed hypoiodite mediated reactions,^20^ asymmetric organocatalysis in drug discovery,^21^ enantioselective organocatalysis in disguise,^22^ asymmetric micheal addition of unactivated ketones and fluorination reaction.^23,24^ In this study, we gave a general overview of the methods and reaction mechanism (Table 1) for obtaining organocatalyzed synthetic organic compounds which have vast industrial applications such as sensors,^25,26^ fertilizers, pesticides,^27,28^ synthetic polymers, cosmetics, and platform chemicals are all utilized to improve people's standards of life.^29,30^

**Table 1:** Summary of discussed organocatalyzed organic reactions

Catalyst	Compound	Solvent	Reaction mechanism	Reaction time	Yield (%)
4,4′-bipyridyl	1–2	C_6_D_6_	Fischer indole	24 h	95–92
	4–5	C_6_D_6_	Deoxygenative reduction	22 h	60–12
Et_3_N	6	1,4-Dioxane	1,4 Addition	24 h	98
Et_3_N hydroiodide	7	Without solvent	Bronsted acid	20 min	83
Cinchona alkaloids	8	Toluene	Amination reaction	48 h	96
	9	CH_3_Cl	Asymmetric Michael addition	48 h	86
Squaric acid	10	Water	Condensation	30 min	96
	11	DCM	Ring-opening polymerization	10 h	98.7
Taurine	12	Water	Cyclo nucleophilic addition	2–3 h	75–90
	13	Water	—	2–3 h	89–90
	14–15	Water	Aldol condensation	30–40 min	98–99
	16	EtOH	Biginelli cyclo-condensation	3 h	99
l-Proline	17	EtOH	Knoevenagel condensation	12 min	94
	18	EIOH	—	10 min	95
	19	EtOH	—	19 min	90
	20	Toluene	Diels–Alder reaction	10 h	82
CPA	21	CH_3_Cl	Atroposelective construction	15 min	87
	22	Toluene	Atroposelective construction	24 h	88
THC	23	DMSO	EDA	14 h	52
	24	DMSO	—	—	84
	25	DMSO	—	—	73
Salicylic acid	26	CH_3_CN	Radical sulfonylation	30 min	67
	27	DMSO	Oxidative condensation	48 h	71
	28	DMSO	Oxidative olefination	24 h	92
DBU	29	DMSO	Cyclo condensation	20 h	92
	30	DMSO	—	20 h	Traces

## 2 Organocatalyzed synthesis and reaction mechanisms

### 2.1. 4,4′-Bipyridyl catalyzed synthesis of indoles

**Scheme 1:**
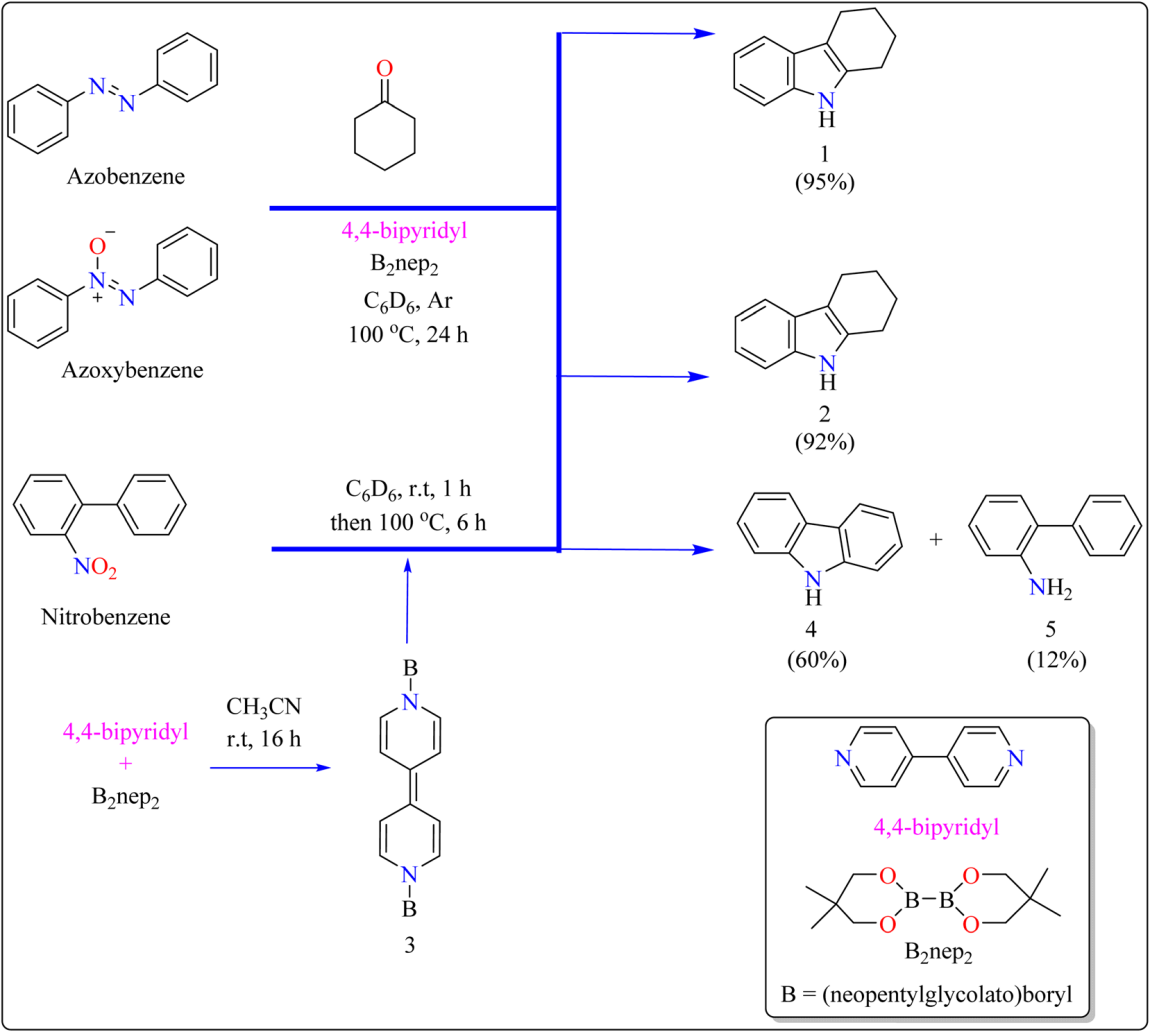
Synthesis of indole at optimized reaction conditions.

Indole is a manufactured desirable structural component that is frequently used in a wide range of organic compounds, including drugs, agrochemicals, dyes, and^31^ the food sector has shown a great deal of interest in red colorant that is an indole-based dye.^32,33^ Indoles with different substituents were synthesized using a variety of simple processes that extensively studied over the past century.^34^ Previously, 4,4′-bipyridyl was widely employed as a rigid linear bridging ligand in the design and fabrication of multidimensional coordination polymers. The literature has various homopolymetallic complexes containing 4,4′-bipyridyl, which is valued for its stiff, stable structure and two divergent binding sites, making it ideal for the formation of coordination networks. Despite the potential rotation of its pyridyl groups, the ligand's value is enhanced by its appropriate length and capacity to generate molecular cavities.^35,36^ However, in recent years, it has additionally been used as an organocatalyst in the synthesis of indoles.^37^ Bis(neopentylglycolato)diboron (B_2_pin_2_) served as a reductant to aid in the *trans*-1,2-diboration of carboxylates using 4,4′-bipyridyl.^38^ Recently, it has been shown that, in the presence of catalytic amounts of 4,4′-bipyridyl, B_2_nep_2_ also acts as a reductant for azobenzene, azoxybenzene, and nitrobenzene, resulting in increased yield and selectivity of indole derivative synthesis (Scheme 1).

**Scheme 2:**
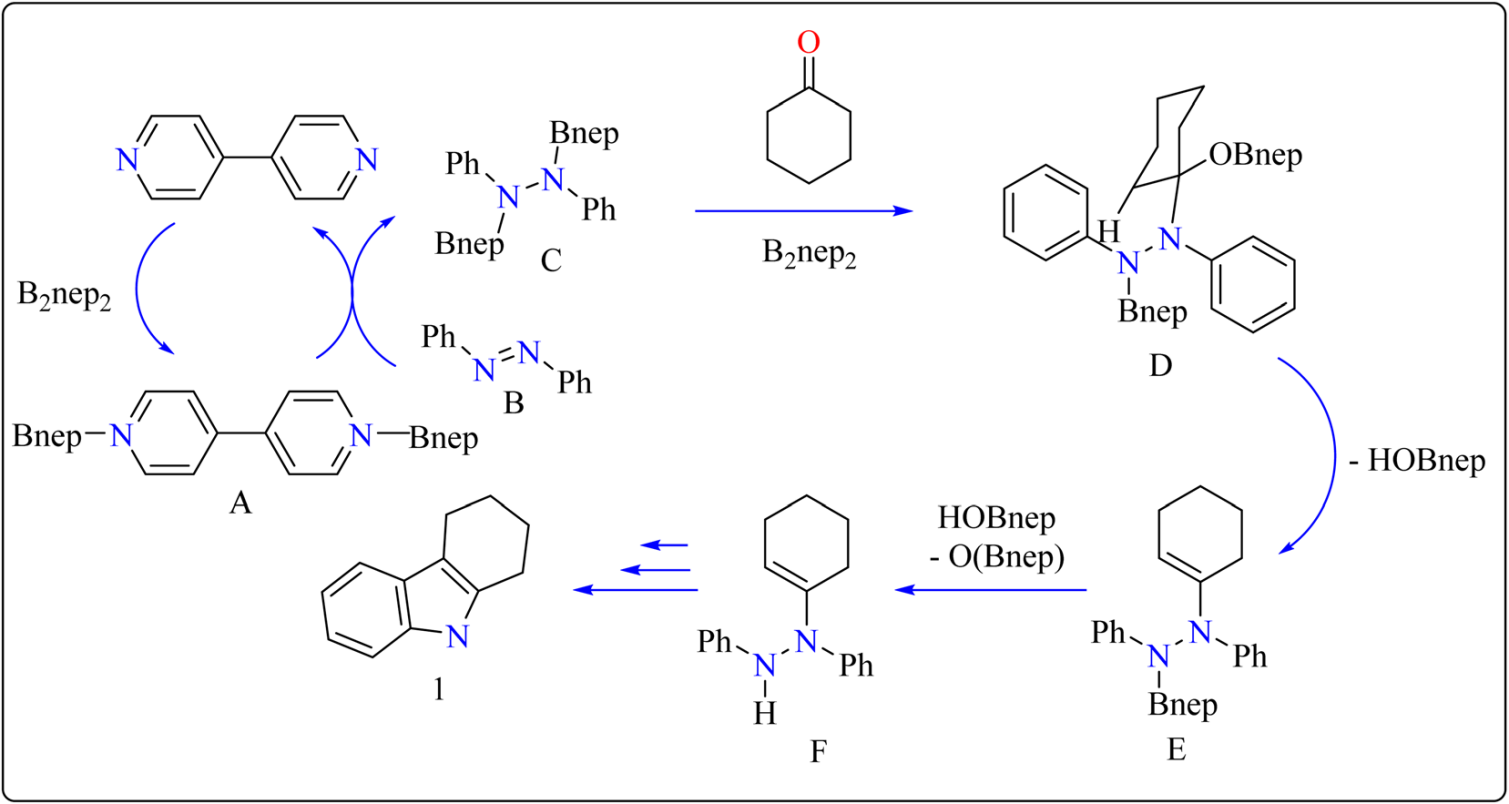
Reaction mechanism for the formation of indole over 4,4′-bipyridial catalyst.

In the synthesis of indole, azobenzene interacts with cyclohexanone in the presence of B_2_nep_2_ (2.2 equiv.) and a catalytic quantity of 4,4′-bipyridial. The reaction was performed in a variety of solvents, including C_6_D_6_, THF, and acetonitrile, in an argon environment at 100 °C for one day, with excellent results. During the first 10 minutes, the tube was gently flipped a few times to dissolve any particulates before being returned to the water at 100 °C. After the reaction was completed, the tube was allowed to cool to room temperature. The reaction mixture was then filtered through Celite and poured directly into a round-bottom flask. The tube was rinsed with ethyl acetate, and the solvent that was used is evaporated to obtain compound 1 with 95% yield.^39^ The same technique was followed with azoxybenzene as a precursor and 3.3 equivalents of B_2_nep_2_, giving compound 2 in 92% yield. For the synthesis of compound 4, 4,4′-bipyridyl interacts with B_2_nep_2_ in CH_3_CN for 16 h at room temperature, yielding intermediate compound 3. This intermediate (3.3 equiv.) subsequently interacts with nitrobenzene in C_6_D_6_, where the reaction mixture was heated to 100 °C for 6 h. This procedure yields two primary products: carbazole compound 4 (60% yield) and compound 5, which yields 12%. Compound 4 was synthesized by inserting the arylnitrene intermediate into the original compound's ortho C–H bond.^40^ The suggested reaction process comprised the diborylation of azobenzene (B) by *in situ*-generated compound A, which yields diborylated compound C (Scheme 2). This interacted with cyclohexanone to produce enamine (F). The atom of nitrogen of compound C reacts nucleophilically with the carbonyl of cyclohexanone, for producing intermediate D and eliminating HOBnep to produce species E. This was quenched by HOBnep, which produced enamine F and O(Bnep)_2_, followed by indole derivative 1 by aniline elimination, analogous to Fischer indole synthesis.^39^

### 2.2. Triethylamine catalyzed reactions

#### 2.2.1. Synthesis of anthrone functionalized benzylic amines

Anthrone and its derivatives are common components in medications, dyes, and optoelectronic materials, as well as natural products and restorative drugs.^41–44^ They have high therapeutic potency, including antibacterial, diuretic, antipsoriatic, telomerase inhibitory, and selective anticancer activity.^45–50^ Functionalized anthrone derivatives can be synthesised by non-asymmetric and asymmetric 1,4-addition, as well as Diels–Alder reactions.^51–54^ While Kitamura *et al.* reported the coupling of 2-hydroxyindoline with anthrone using BF_3_·Et_2_O,^55^ and White's group established a two-step technique employing Fe(PDP)-catalyzed oxidation followed by 1,2-addition of anthrone,^56^ the 1,2-addition of imine remains undiscovered. Recently, researchers reported for the first time a Et_3_N direct 1,2-addition of anthrone to *N*-sulfonylaldimines over moderate conditions, resulting in benzyl amine functionalized anthrone.

**Scheme 3:**
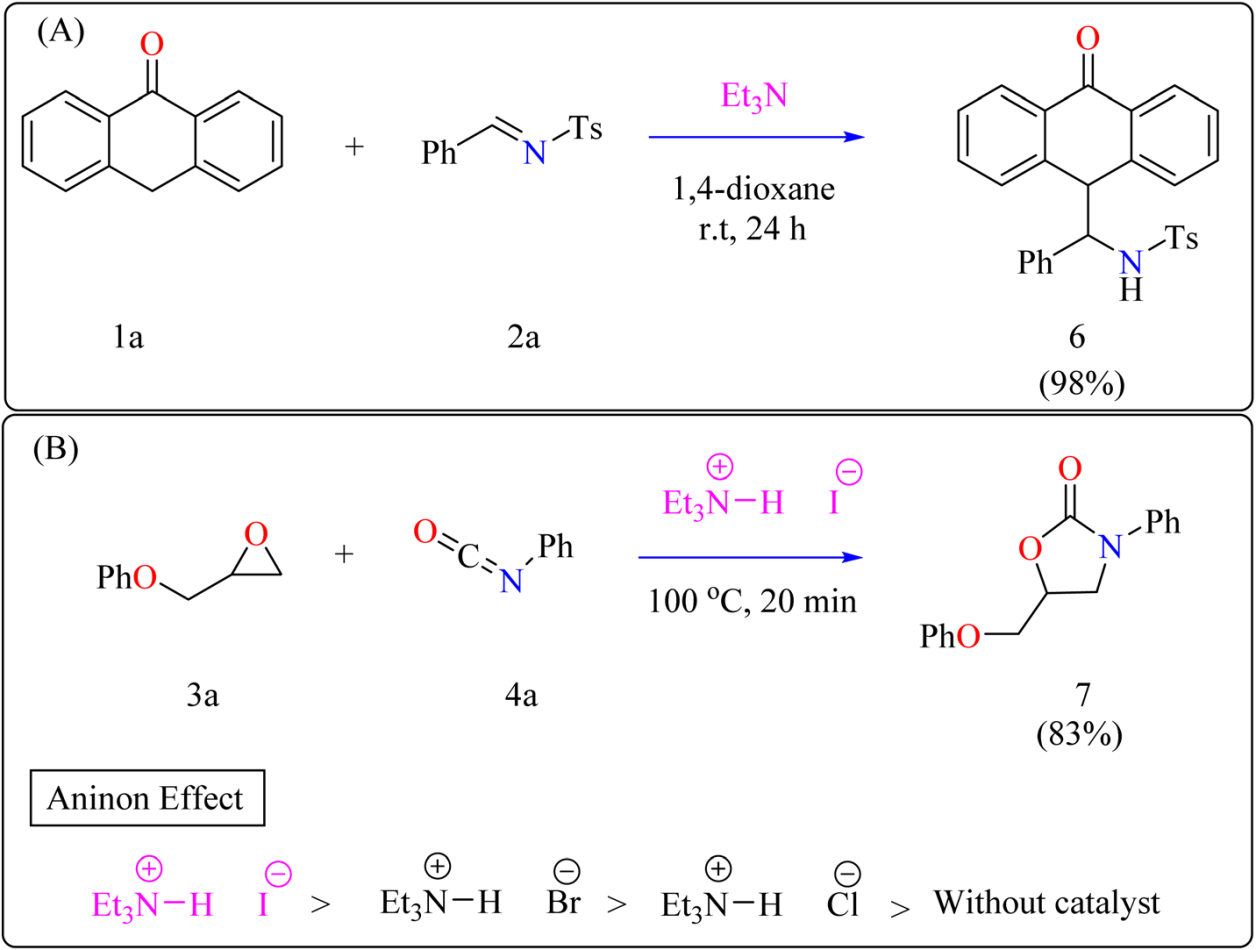
Et_3_N catalyzed synthesis of (A) Anthrone functionalized benzylic amines and (B) 2-oxazolidinones.

Anthrone 1a, *N*-benzylidene-*p*-toluenesulfonamide 2a, and catalytic amount of Et_3_N (20 mol%) were mixed in an oven-dried round-bottom flask with anhydrous 1,4-dioxane, flushed with N_2_, and agitated for 24 h at room temperature (approx. 32 °C). The crude reaction mixture was then concentrated over reduced pressure and refined directly *via* silica gel column-chromatography, using petroleum ether/DCM/EtOAc as eluent, providing compound 6 with a 98% yield (Scheme 3A). Thus, Et_3_N was shown to be the best catalyst, yielding the desired product in 98% of the reactions. However, an observational study showed that the organocatalyst plays a significant part throughout the 1,2-addition process because the reaction only produced a little amount of product in its absence.^57^

#### 2.2.2. Synthesis of 2-oxazolidinones

Penta heterocycles, notably 2-oxazolidinones (7), are important in drug design, with several medications, like the anticoagulant rivaroxaban, built around this core.^58–61^ Previous literature demonstrated viable methods for synthesizing five-membered cyclic carbonates utilizing epoxides and carbon dioxide *via* the use of Et_3_N hydroiodide as an excellent bifunctional catalyst.^62–66^ The catalytic activity of Et_3_N hydroiodide, renowned for its acidic hydrogen on the atom of nitrogen, was investigated for coupling epoxides and isocyanates to produce 2-oxazolidinones. This simple and affordable catalyst acts as a bifunctional agent, effectively facilitating the necessary reactions.^67,68^

**Scheme 4:**
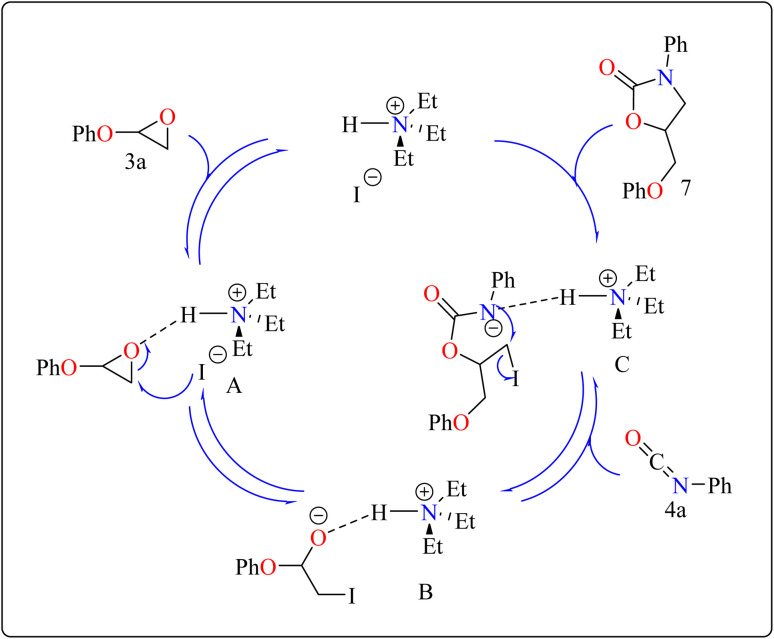
Schematic route for the Et_3_N catalyzed synthesis of compound 7.

Developing effective methods for the synthesis of compound 7 is critical.^69,70^ Recent research shown that Et_3_N hydroiodide can efficiently catalyze the coupling of epoxides and aryl isocyanates and produced compound 7 without any byproduct. The reaction takes place under basic conditions: glycidyl phenyl ether 3a, phenyl isocyanate 4a, and Et_3_N hydroiodide (10 mol%) are exposed to heat to 100 °C (Scheme 3B). After 20 minutes of stirring, the goal product 7 is achieved in 83%. The catalytic efficiency of ammonium salts varies with halide anions; iodide is the most efficient, generating 7 in 83%, while bromide and chloride yield 44% and 12%, respectively. Previous experiments involving cyclic carbonate synthesis have shown that no product formed without a catalyst. According to Scheme 4, the catalytic procedure using Et_3_N hydroiodide includes epoxide 3a activation *via* hydrogen bonding with the catalyst (intermediate A).^62^ The activated epoxide is nucleophilically attacked by an iodide anion, resulting in intermediate B. This intermediate's alkoxide anion subsequently attacks 4a, resulting in intermediate C. The target product 7 is produced through intramolecular ring-closing of C, which also regenerates the catalyst. The iodide anion's efficacy stems from its low coordination with hydrogen that is acidic and high leaving group ability.^71^

### 2.3. Cinchona alkaloid catalyzed reactions

Cinchona alkaloids (quinine) have emerged as key asymmetry inducers in enantioselective catalysis over the last decade. These compounds are effective agents because of their structural characteristics, particularly a strong and easily functionalize chiral framework.^72^ Cinchona alkaloids are a truly green commodity because they are obtained from biomass and are renewable resources. They have been used as chiral Lewis bifunctional catalysts, chiral bases, and quaternized ammonium salts are utilized in phase-transfer catalysis for the past 20 years.^73,74^

#### 2.3.1. Synthesis of pyrazolones

The pyrazolones, classified as nitrogen-containing five-membered heterocyclic compounds, are frequently utilized as synthetic substrates in chemical and pharmaceutical chemistry as well as chelating, coloring, and pharmacological agents.^75–79^ A common pyrazolones interaction is the synthesis of heterocyclic dyes having long conjugated systems.^80^ Fascinatingly, prior to 2008, there were no organocatalytic methods for the enantioselective production of functionalized pyrazolones.^81^ In 2009, Zhao published the initial instance of enantioselective reactions organocatalyzed by utilizing pyrazolone analogues. After this different organocatalysts (Thiourea, Gadolinium) used to catalyze enantioselective reactions.^82^ Here discussed quinine as organocatalyst for amination of pyrazolones. Quinine has been noticed as an appropriate catalyst and showed enantioselectivity up to 97%.

**Scheme 5:**
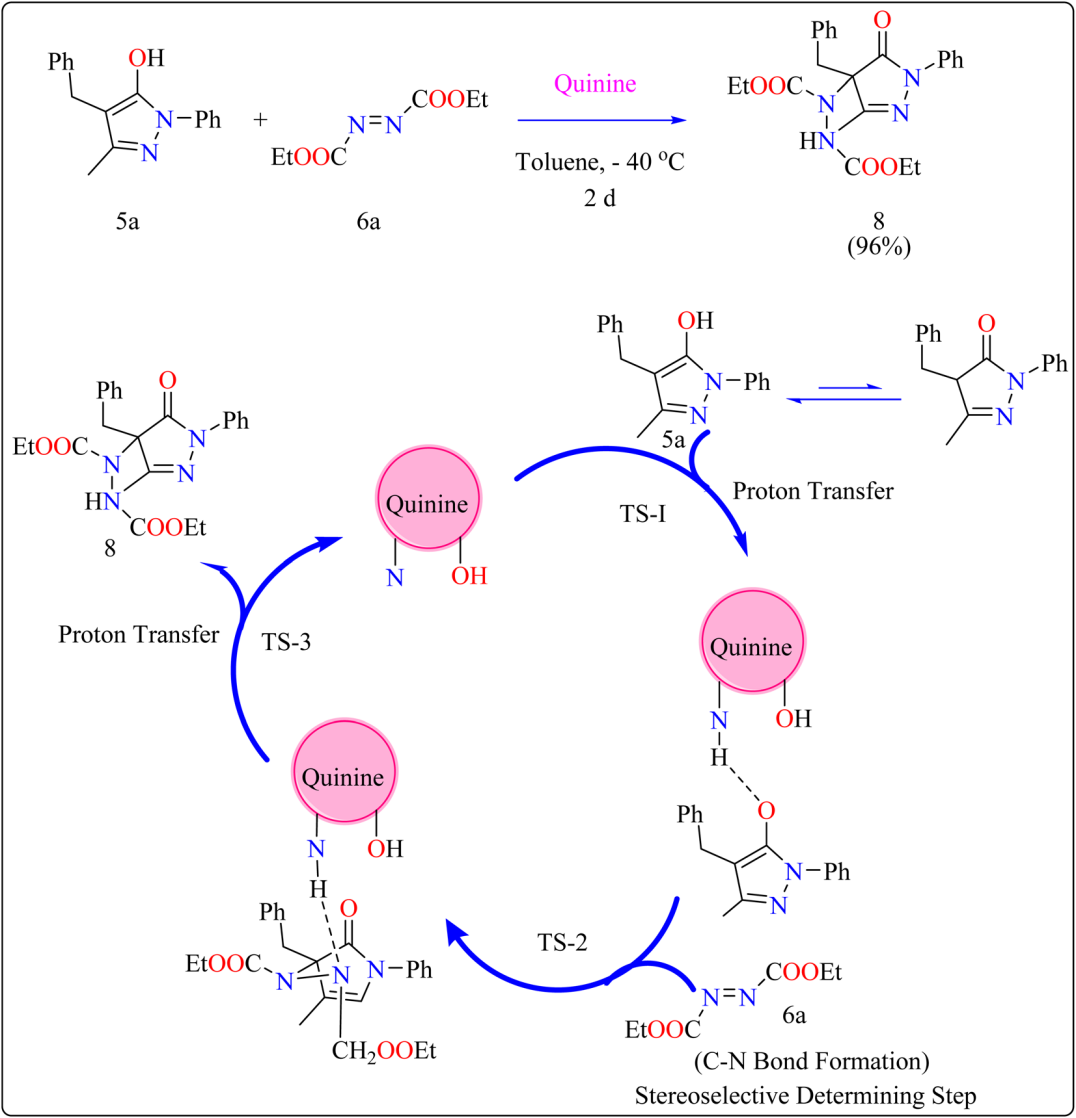
Synthesis and proposed reaction mechanism for quinine catalyzed compound 8.

**Fig. 1:**
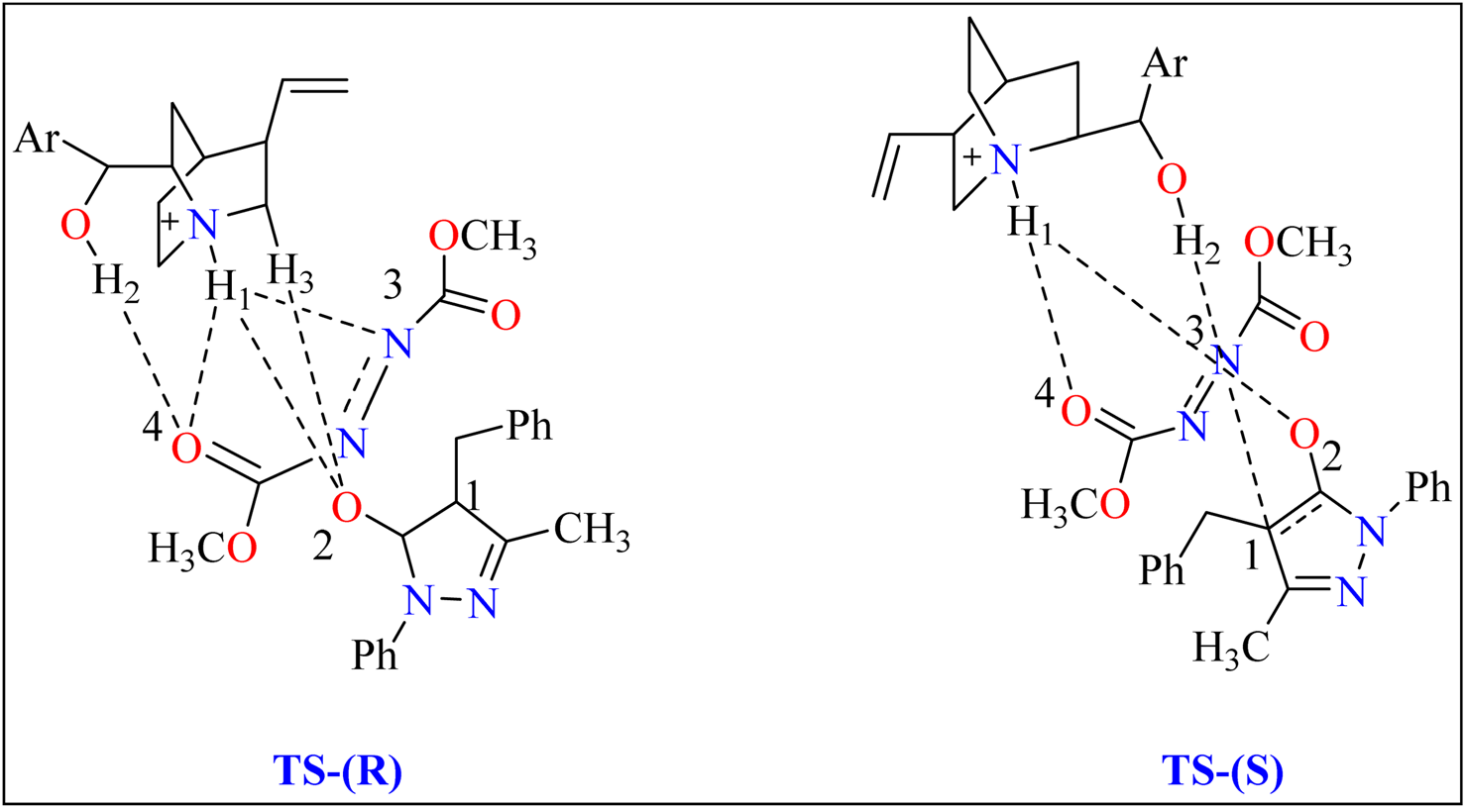
Representation of two significant transition states of compound 8, TS-(R) and TS-(S).

General procedure for the synthesis of functionalized pyrazolones proceeded with substituted pyrazol-5-one 5a and quinine mixed in 2 mL of toluene and agitated for ten minutes at ambient temperature (Scheme 5). The reaction mixture was cooled to −40 °C. The next step involved adding azodicarboxylate 6a, stirring was done in the reaction mixture. The reaction mixture was agitated at −40 °C until complete conversion. After that, a silica gel column directly loaded with the reaction mixture. Column chromatography (hexanes/EtOAc) produced the desired product 8. When quinine is used to catalyze the reaction, pyrazolones might undergo the catalytic mechanism as shown in Scheme 5. In initial step pyrazolone deprotonated and proton transferred to catalyst, in second step added to azodicarboxylate, carbon–nitrogen (C–N) bond formation step which predicted to serve as the reaction's step that determined stereoselectivity, and at the last proton transferred from the catalyst to adduct. As seen in (Fig. 1), the optimized transition states of pyrazolone. The ‘R’ product was produced when pyrazolone (Re-face) attacked by azodicarboxylate through TS-(R), while ‘S’ product was produced when pyrazolone (Si-face) added to azodicarboxylate *via*TS-(S). There are three key interactions based on H-bonding with (catalyst)NH⋯N(azodicarboxylate), (catalyst)NH⋯O(pyrazolone), and (catalyst)OH⋯O(azodicarboxylate) were found to be effective for charge stabilization on transition states. In addition, the frameworks made of pyrazolone and azodicarboxylate also take advantage of extra stabilization, bonded *via* hydrogen interactions in the TS-(R).^83^

#### 2.3.2. Synthesis of chiral maleimide

Maleimide derivatives have excellent physical and biological features, making them suitable frameworks.^84–87^ Although extensively investigated as Michael acceptors in asymmetrical Michael addition, which produces succinimides, their application as nucleophiles is uncommon.^88,89^ Some operations have used maleimides to produce maleimide-containing compounds by directly introducing the maleimide moiety. However, reactions using α-aminomaleimides as asymmetric Michael donors have not yet been documented. This study demonstrated the first asymmetric Michael addition of α-aminomaleimides (8a) as Michael donors to β-nitrostyrenes (7a) utilizing a bifunctional quinine organocatalyst. This expands the nucleophilic uses of maleimides.

**Scheme 6:**
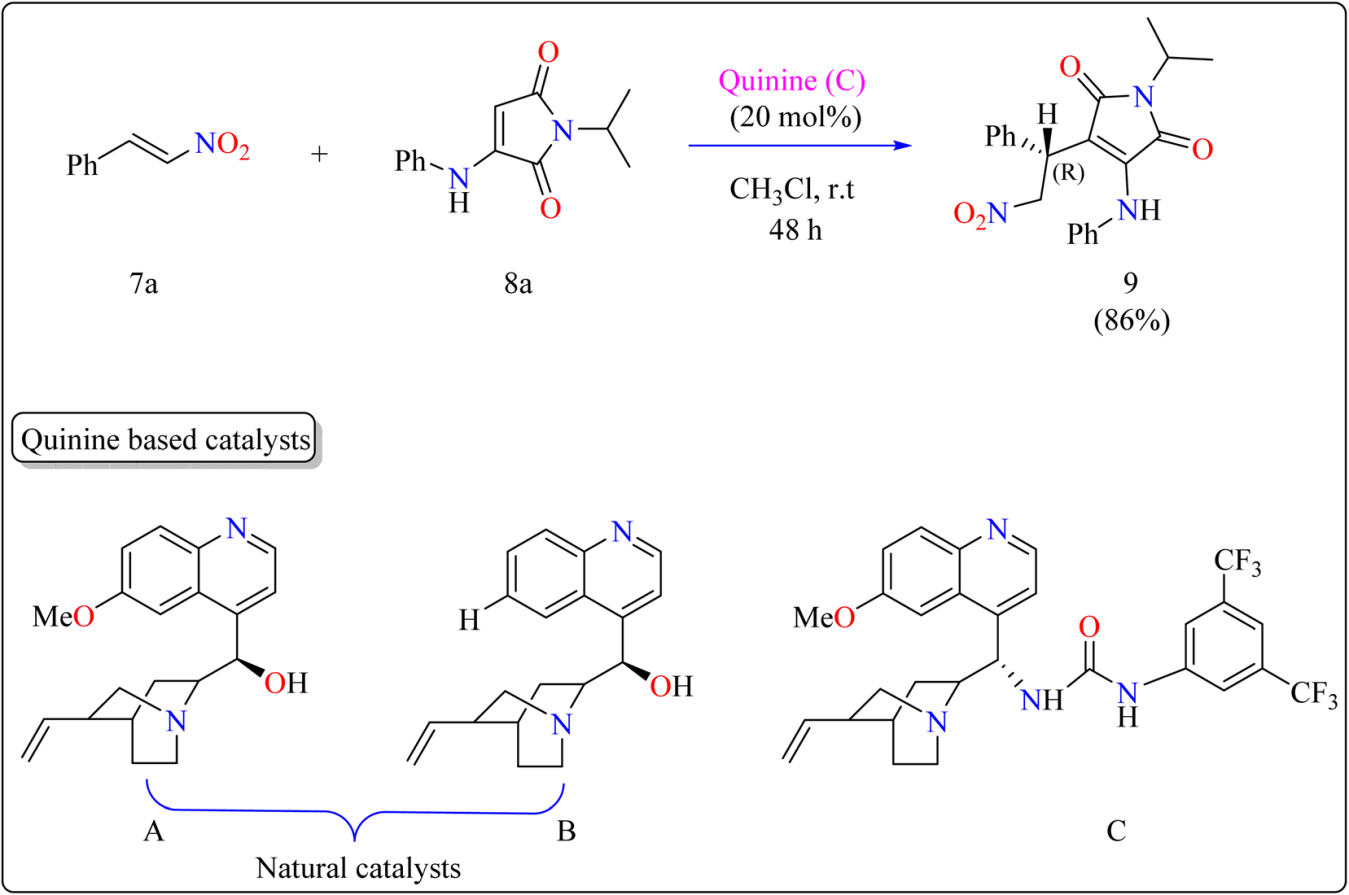
Quinine catalyzed synthesis of maleimide.

Researchers used 7a as a Michael acceptor to optimize the organocatalyst. Using natural quinine and cinchonidine, they were able to produce the Michael adduct 9 in moderate to good yield. However, the use of benzoyl-protected quinine decreased the yield. The incorporation of urea and thiourea molecules increased yield even further. Notably, C catalyst, generated from quinine and containing a 3,5-bis(trifluoromethyl)phenyl moiety, obtained 86% yield and 88% enantioselectivity, making it the best catalyst. As shown in Scheme 6, the reactions were conducted under the following circumstances: The substrate 7a and 8a were mixed in equimolar concentrations (0.1 mmol each), containing 20 mol% catalyst C in 1 mL of CHCl_3_. The combinations were swirled at ambient temperature for two days in an open-air setting.^90^

### 2.4. Squaric acid catalyzed reactions

Squaric acid (SA), a dibasic organic acid which has a decomposition temperature of 245 °C, functions as a green organocatalyst.^91^ Due to resonance stabilization, it is very acidic in water (p*K*_a_ = 1.5, 3.4). It is soluble in water while insoluble in the majority of organic solvents.^92^ This acid has been used in organic transformations, including the production of *N*-substituted pyrroles, bis(indolyl)methanes, Mannich reactions and Michael additions.^93–96^

#### 2.4.1. Synthesis of perimidines

Perimidine derivatives, which are key nitrogen-containing heterocycles, have a variety of biological and pharmacological actions, including antibacterial, antimicrobial, anticancer, and anti-inflammatory characteristics.^97–99^ Their high electron-donating properties make them useful intermediates in the synthesis of symmetrical squarylium dyes for NIR absorption. In addition, they serve as antioxidant stabilizers, photochromic chemicals, catalysts, ligand scaffolds, and supramolecular stoppers.^100–103^

**Scheme 7:**
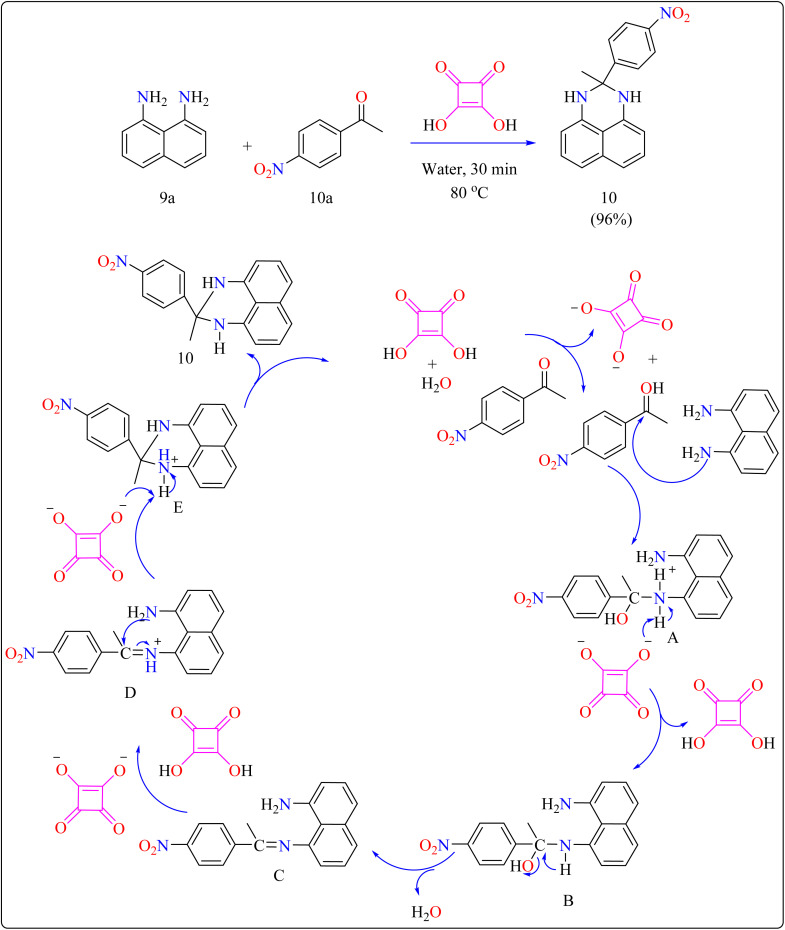
Synthetic route for the squaric acid catalyzed synthesis of perimidines.

Researchers devised a green synthesis technique for 2,3-dihydro-1*H*-perimidines (10) that utilizes squaric acid as an organocatalyst and water as the solvent. This innovative method comprises heating a combination of 1,8-diaminonaphthalene (9a), acetophenone (10a), and squaric acid (10 mol%) in 5 mL of water at 80 °C (Scheme 7). After the reaction is finished, the mixture was allowed to cool and the products are purified with filtration and water washing. Based on literature,^104,105^ predicted procedure indicates that squaric acid's strong acidic aids during the condensation of compound 9a and 10a, resulting in a Schiff's base intermediate. This intermediate is then protonated and cyclized within the same molecule. A second proton abstraction by squaric acid's dianion yields the final product 10 as well as regenerates the squaric acid catalyst, allowing it to be reused. This approach is significant for its use of water as a sustainable solvent and the catalyst's recyclability. The researcher investigated the substrate breadth of this method using different ketones and compound 9a. Aliphatic and alicyclic ketones reacted faster and produced more compounds than aromatic ketones, possibly due to steric hindrance. Electron-deficient aromatic ketones, such as 10a containing nitro group, finished the process in 30 min resulting in a 96% yield, whereas electron-rich ones, such as 4-methoxy acetophenone, required 105 min with an 86% yield.^106^

#### 2.4.2. Synthesis of polyester

Here reported, SA was meticulously assessed for its catalytic performance when utilized as an acidic organocatalyst to perform the ring opening polymerization of cyclic lactones or carbonate. In general, the scientists focused on synthesizing effectively designed polyester poly(caprolactones) (PCLs). Regarding the rising demand for environmental and safety, instead of the time-consuming and expensive heterogeneous catalyst manufacturing process, SA might be employed as a directly renewable and recyclable organocatalyst toward the manufacturing of biodegradable and biocompatible polyesters. PCLs is a structural component of Indolinospiropyran-poly(e-caprolactone) dye films which converted into merocyanine dye by applying mechanochemical strain.^107^ Adjusting the amount of monomer, the polymer length is investigated, so light intensity and photostability are enhanced when the polymer's ideal molecular weight prevents fluorescence quenching. Because of their possible applications in electronic devices, photonics, optical technology, chemosensors, and photonics, organic luminous compounds have become fascinating for industry and research.^108,109^

**Scheme 8:**
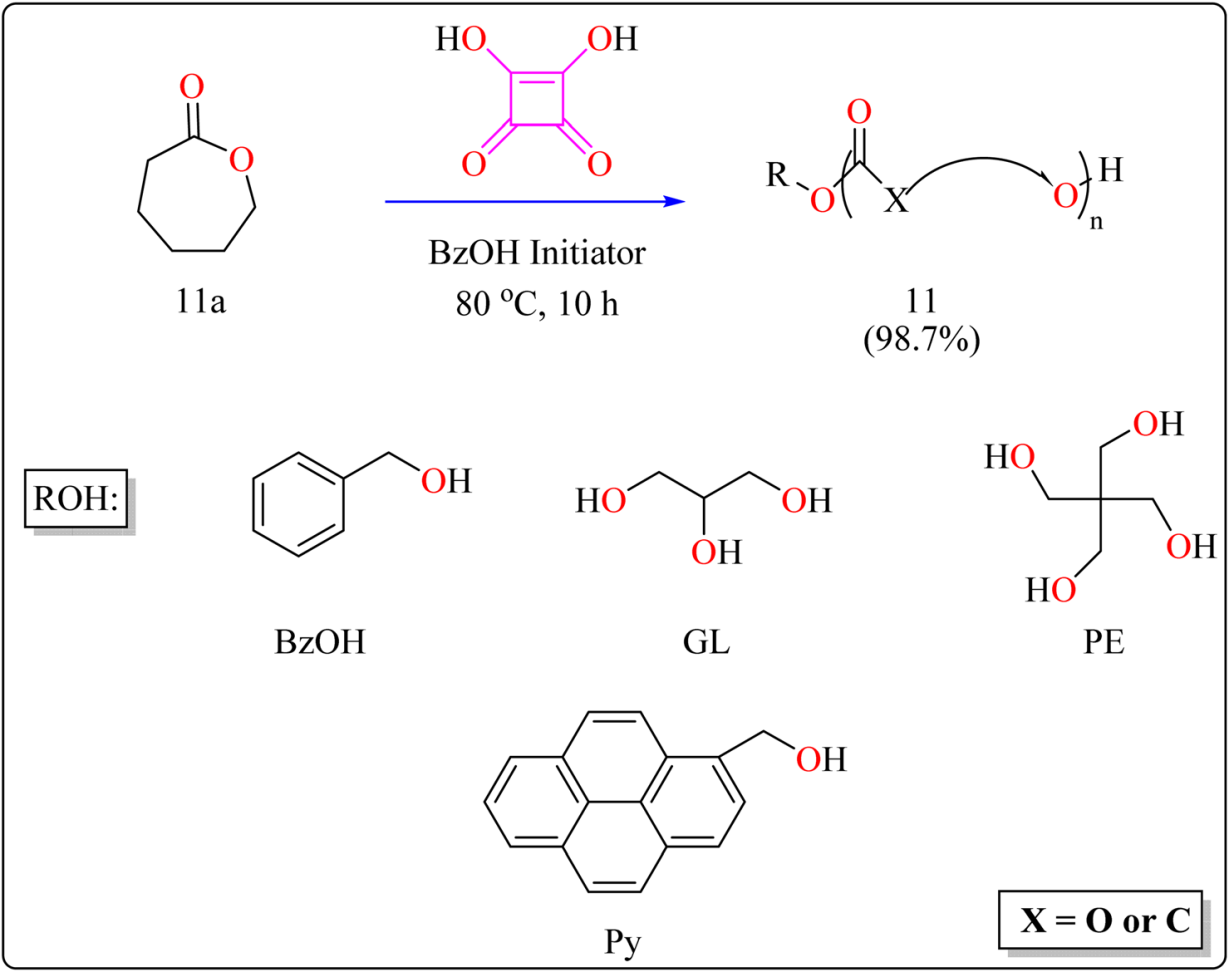
Synthetic of polyester.

**Scheme 9:**
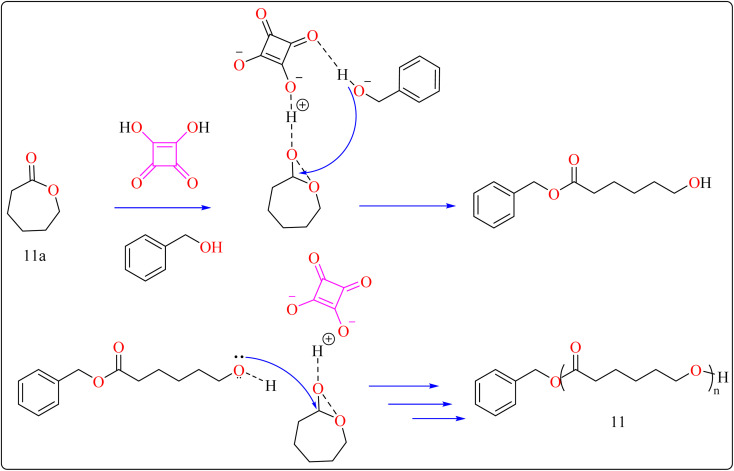
Mechanism for squaric acid catalyzed synthesis of poly(ε-caprolactones).

The following substances were put in to a Schlenk containing a stirrer that was magnetic in the glove compartment: caprolactones (11a), BzOH initiator, and SA catalyst (1.0 equiv.) (Scheme 8). The polymerization then finally quenched by adding Amberlyst A21 for the specific time while the reactor was set at 80 °C. A specified amount of the polymerization mixture withdrawn. Dichloromethane (DCM) added to the reaction mixture after the allotted time to dissolve the polymerized PCL, and the SA catalyst has been removed by filtering. The filtrate was ultimately vacuum-evaporated in order to generate white solid of PCL 11. Simply filtration, washing, and drying were used to isolate SA for each catalytic cycle. Interesting is that SA can be easily retrieved and utilized again for more than ten successive cycles without noticeably demolish its catalytic activity.^109,110^Scheme 9 showed the catalytic pathway for the synthesis of aliphatic polyester 11.

### 2.5. Taurine catalyzed reactions

Taurine (2-aminoethanesulfonic acid), a commonly available amino acid that is also cheaply priced, has recently been employed to accelerate several chemical reactions.^111,112^ In nature, taurine is a common S-containing amino acid that can be obtained from a variety of living things.^113^ Due to the presence of the –SO_3_H group, taurine, which is widely accessible and moderately priced, has the ability to function as proton-acid catalysts in the process of oxidation of sulphides towards sulfoxides utilizing aquatic peroxide of hydrogen to act as oxidant. Taurine has important biological roles in the body and is a key ingredient in energy drinks and supplements. Furthermore, it promotes the Knoevenagel reaction in aqueous solution and helps for synthesizing derivatives of barbituric and thiobarbituric acid.^114–116^

#### 2.5.1. Synthesis of spirooxindole dihydroquinazolinones (12) and novel 1,2-dihydroquinazolinones-3(4*H*)isonicotinamides (13)

**Scheme 10:**
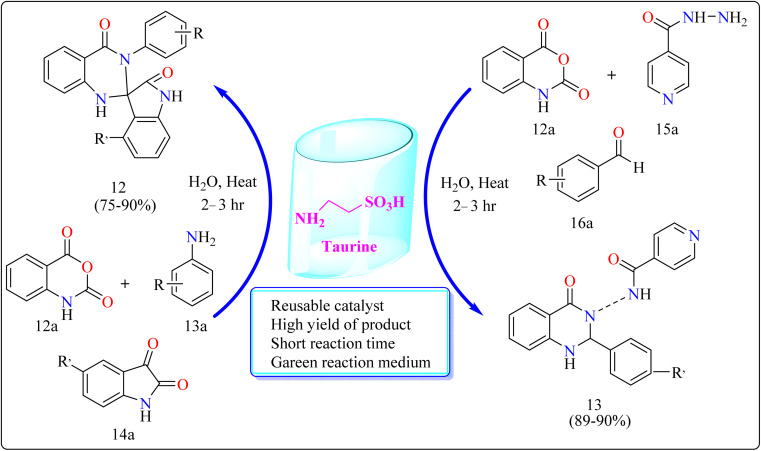
Methodology for the synthesis of compound 12 and 13.

The researchers evaluated the reusability of taurine for each of the reactions comprising compound 12 and 13. Without significantly affecting the product yield, it was simple to recover and reuse the 2-aminoethanesulfonic acid for at least three runs. Taurine catalyzed synthesis of 12 and 13 showed in Scheme 10. Taurine catalyst (15 mol%) was introduced to an equimolar mixture of isotonic anhydride (12a), aniline (13a), and isatins (14a) for the reaction, took 10 mL of H_2_O inside a 100 mL round-bottom flask. For a period of six hours, the reaction mixture had been heated. After the reaction, that was monitored by TLC, finished, 10 mL of water was added to it and the mixture was stirred for 3 minutes. Then, it was allowed to cool. At this point, the material precipitated, and it was later separated by filtration. The isolated product had many water rinses. No further processing using column chromatography as well as the utilization of any kind of organic solvent was necessary after drying to produce the pure product. Here excellent yield of product was obtained in ranging from 75–90%. Similarly for the synthesis of compound 13. Taurine catalyst (15 mol%) was introduced to an equimolar mixture of 12a, isoniazid (15a) and aldehyde (16a) in 10 mL of water in a round bottom flask for the reaction. The reaction mixture was heated for 2.5 h. Next procedure for the synthesis of VIIC same as used for the synthesis of IVC. The product yield was obtained in the range of 89–90% (Scheme 10). Quinazolinone derivatives have multiple applications in the dyestuff enterprises as coloring ingredients in addition to their use in pharmaceuticals.^117,118^

**Scheme 11:**
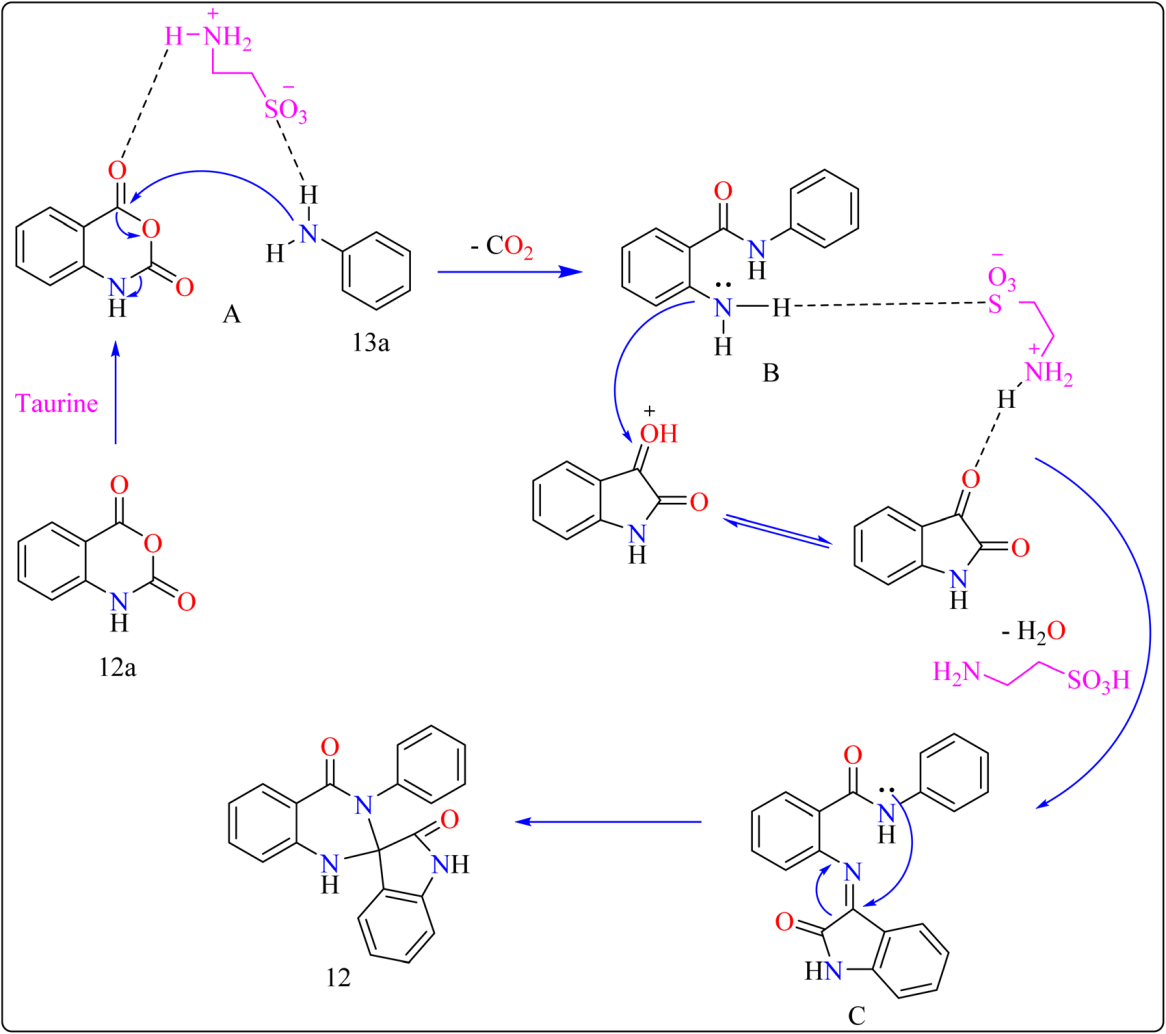
Reaction mechanism for the synthesis of taurine catalyzed compound 12.

Reaction mechanism for the synthesis of compound 12 showed in Scheme 11. Here taurine functions as a donor–acceptor reagent with dual functions by activating 12a carbonyl position to produce intermediate A, which may allow 13a nucleophilic attack on the carbonyl unit. 13a was added nucleophilically to 12a, which was then decarboxylated to form 2-aminobenzamide (B). Imine intermediate C was then produced by condensation of B with protonated isatin, which was made using taurine, and then underwent intramolecular cyclization to produce product 12.^119^

#### 2.5.2. Synthesis of 3,4-dihydropyrimidin

**Scheme 12:**
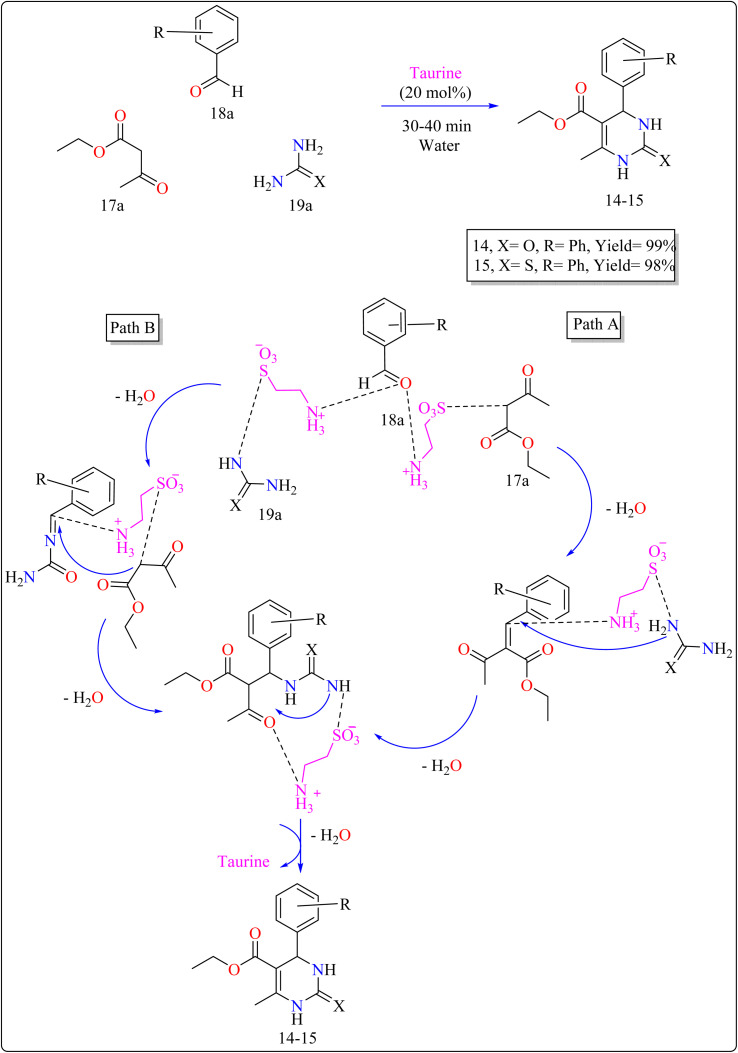
Synthesis and proposed mechanism for taurine catalyzed compound 14–15.

The use of taurine as a green bio-organic catalyst enhances the Biginelli reaction, a one-pot multicomponent synthesis of bio-active 3,4-dihydropyrimidin-2(1*H*)-ones/thiones (14–15). This method employs ethyl acetoacetate (17a), an aromatic aldehyde (18a), and urea or thiourea (19a) in an aqueous solution of EtOH (1 : 3) as shown in Scheme 12. The reaction produced high yields, is simple, and environment friendly, with short reaction times and minimal costs. Without a catalyst, the process produces little product. Several catalysts (acetic acid, l-proline, *p*-toluenesulfonic acid, FeCl_3_·6H_2_O) were tried, and taurine produced the greatest yields and quickest reaction durations, especially at 20 mol%. Raising the total amount of taurine failed to considerably improve yield. Solvent investigations showed that a water–ethanol mixture outperformed other solvents such as DCM, EtOH, DMF, MeOH, 1,4-dioxane, MeCN, toluene, and pure water, all of which produced lower yields. The technique advantages from fast completion and easy product purification using ethanol recrystallization, thereby providing an effective and environmentally benign approach for synthesizing compound 14 and 15.^120^ In Scheme 12, two approaches are presented on the basis of previous studies^115^ for synthesizing 14–15 products with taurine as a catalyst. Pathway A involves taurine promoting aldol condensation of 17a and 18a, followed by nucleophilic addition of 19a and hydrogen shift to produce compounds 14–15. Pathway B begins with the nucleophilic addition of 19a to 18a, followed by taurine-catalyzed imine production, and then addition and cyclization with 17a.^120^

**Scheme 13:**
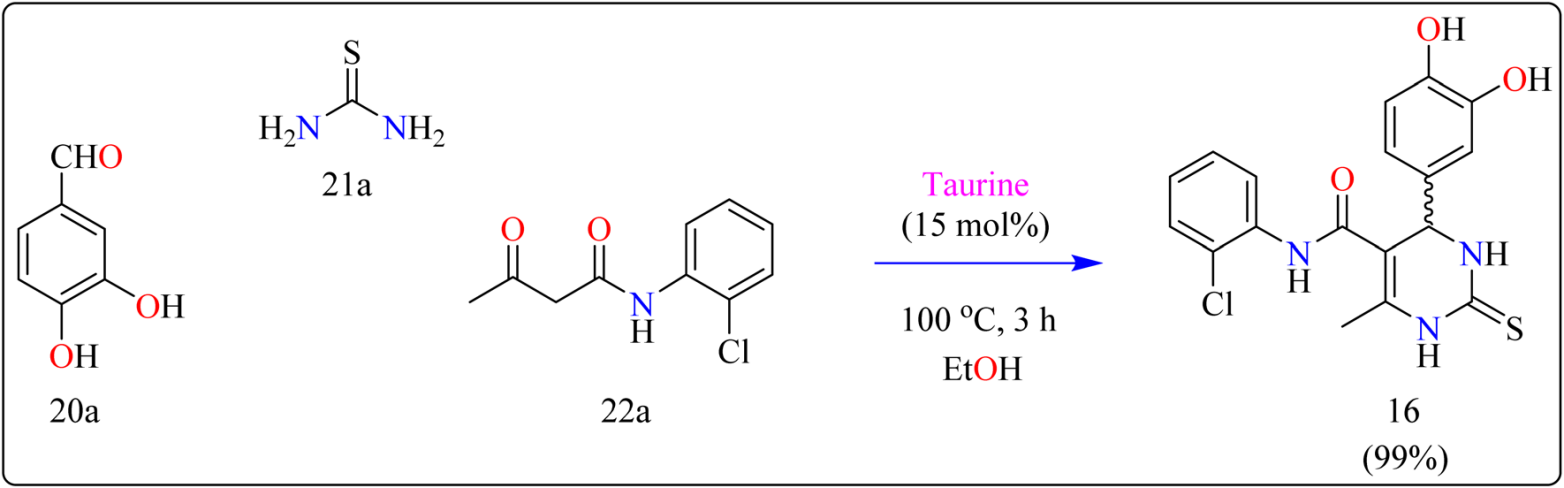
Synthesis of taurine catalyzed compound 16.

Compound 16 was synthesized using important precursors such as substituted benzaldehyde (20a), thiourea (21a), and acetoacetanilides (22a). The reaction, which was carried out in ethanol at 100 °C, took 3 hours to complete (Scheme 13). The use of taurine as a catalyst considerably increased the efficiency of the reaction, generating compound 16 with a high productivity of 99%. This one-pot synthetic approach not only produces excellent yields but also streamlines the process, making it appropriate for large-scale industrial manufacturing.^121^ The usage of taurine corresponds with environmentally friendly techniques, increasing the method's overall sustainability. This synthesis's efficiency and speed, together with its high yield, make it a valuable technique for synthesizing 3,4-dihydropyrimidin derivatives, which have been shown to be bioactive. The fact that this technology can be applied to large-scale production highlights its potential for wider industrial adoption.^122^

### 2.6. l-Proline catalyzed reactions

l-Proline, a naturally occurring amino acid having secondary amine functionality, operates as a bifunctional organocatalyst, with the amino group acting as a Lewis base and the group of carboxylic acids serving as a Brønsted acid.^123–125^ It is a highly efficient, adaptable, and environmentally friendly catalyst for the synthesis of heterocyclic systems. l-Proline efficiently catalyzes a variety of asymmetric syntheses, such as Ullmann, Hantzsch, and Knoevenagel reactions, in addition to, aldol, Mannich, Robinson annulations, Michael, α-selenenylation, and Diels–Alder. Its dual role as a ligand and catalyst, combined with its excellent enantiomeric purity, increases its value in green chemistry.^126–128^

#### 2.6.1. Synthesis of benzil bis-hydrazones

**Scheme 14:**
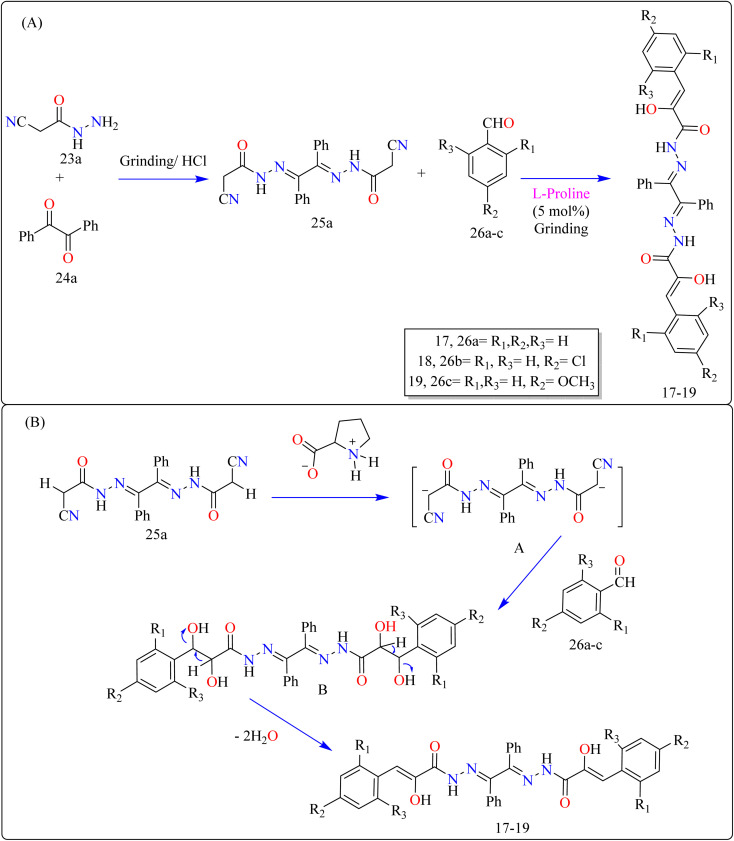
(A) Methodology and (B) proposed route for the synthesis of l-proline catalyzed benzil bis-hydrazones.

Novel bioactive benzil bis-hydrazone derivatives (17–19) were synthesized by solvent-free mechanical grinding of benzil bis(cyanoacetylhydrazone) (25a) with aldehydes (26a–c) employing l-proline as an effective organocatalyst (Scheme 14). The precursor 25a was synthesized by heating benzil (23a) and 2-cyanoacetohydrazide (24a) in EtOH with HCl. The grinding method at room temperature with moist l-proline (5 mol%) produced high yields *via* Knoevenagel condensation. After assessing the completeness of the reactions after 10 minutes of grinding, researchers discovered that yields for compounds 17 and 19 declined from 94% to 83% and 90% to 89%, respectively, while yield for compound 18 remained constant. This shows that electron-donating and electron-withdrawing groups speed up the process, preventing major yield losses. In contrast, the yield of the unsubstituted derivative 17 dropped dramatically, showing a reduced reaction efficiency in the absence of substituents. Thus, substituent groups are critical for maintaining good yields during shorter reaction durations.^129^

#### 2.6.2. Synthesis of functionalized phthalimides

In the manufacture of beneficial substances including catalysts, dyes, polymers, along with fluorescence probes, derivatives of phthalimides are widely employed. The primary use of phthalimide structure in dispersed dyes is as a diazo or coupling component.^130,131^ More Effective coplanar aromatic rings are another feature of phthalimide disperse dyes. Increasing the plane shape of dye molecules is one of the best ways to improve their washing fastness because they result in higher intermolecular interaction energy, decreased temperature migration, and exceptional washing fastness.^132,133^

**Scheme 15:**
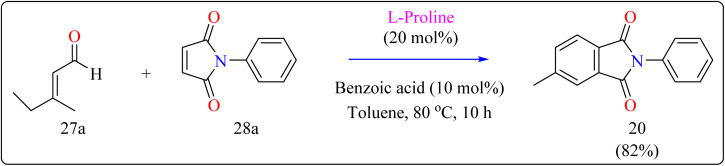
Optimized reaction conditions for l-proline catalyzed compound 20.

Researcher presented the Diels–Alder reaction as a method for the l-proline-catalyzed benzannulation of different and multifunctionalized phthalimides using two dienophiles instead of a diene and a dienophile (Scheme 15). This reaction allowed the transition-metal-free preparation of multiple phthalimides by an organocatalytic method involving maleimides and α,β-unsaturated aldehydes. Here, with the addition of an l-proline catalyst, researcher used a number of additives (acetonitrile, acetic acid, pivalic acid, and benzoic acid) as well as a variety of solvents (THF, dioxane, and toluene). Fortunately, compound 20 was produced in a fast reaction time (10 h) with batter yield of 82% using benzoic acid (10 mol%) as additive, with toluene serving as the solvent. The same way, l-proline and benzoic acid were added to a solution of 3-methyl-2-butenal (27a) and *N*-phenylmaleimide (28a) in toluene. The mixture was agitated for ten hours at 80 °C in an oil bath. When the reaction was determined to be finished by TLC, the product of the reaction had been evaporated using an evaporator with a rotary blade. Applying ethyl acetate/hexane (1 : 20) as an eluent, the residue was subsequently filtered with a silica gel column chromatography technique, producing the desired product 20 in 82% yielding as a yellow solid.^134^

### 2.7. Axillary phosphoric acid catalyzed reactions

#### 2.7.1. Synthesis of axially chiral 4-azaborinephenols

Asymmetric catalysis and materials science rely heavily on axially chiral components found in chiral ligands and organocatalysts.^135^ Research on heterobiaryl atropisomers particularly those with non-C–C or C–N axes is scarce compared to the vast study of biaryl atropisomers.^136–140^ To increase their use in the development of drugs and materials design, innovations in their preparation and characterization are necessary. A desymmetrization approach was taken,^141^ building on the understanding of atroposelective synthesis and effective electrophilic aromatic substitution.^142–144^ A 3,5-disubstituted phenol (29a) motif model substrate was developed. While methyl groups maintained the stereogenic axis for improved enantioinduction, hydroxyl groups served as directing groups and catalyst binding sites.^145^

**Scheme 16:**
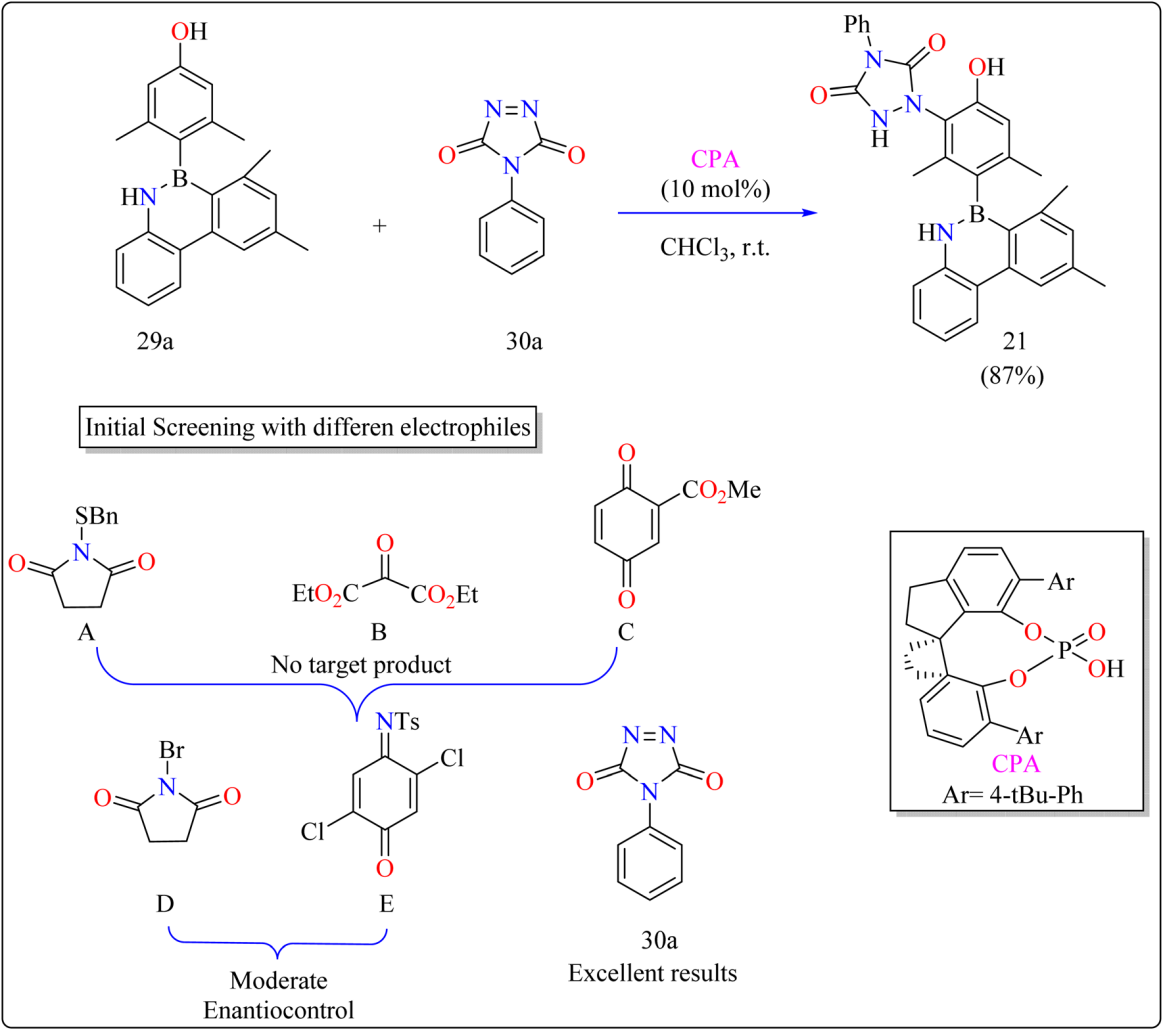
Methodology for the synthesis of CPA catalyzed compound 21.

The difficulties were in finding a chiral organocatalyst that was appropriate for distant axial enantiocontrol, choosing an electrophilic reagent that was effective with phenol, and creating gentle reaction conditions to maintain stability. It was suggested to use CPAs for bifunctional activation through H-bonding interactions. Limited reactivity was seen in the initial substrate reactions with certain electrophiles. A close-by binding site is crucial for stereoselectivity, as demonstrated by the modest enantioselectivities of bromination and a particular reagent (E). Due to its advantageous qualities, diazodicarboxamide 30a which is reactive and compatible with CPA in terms of H bonds was selected. As shown in Scheme 16, in a 15 minutes reaction with 29a, 30a, and CPA (0.01 mmol) in 2 mL of CHCl_3_ at room temperature, 87% of 21 was produced. Even after being heated to 120 °C for 24 hours, the product showed good configurational stability and no discernible racemization. This method effectively transfers stereochemical data obtained from the catalyst to the B–C linkage of azaborine derivatives by remotely setting the stereogenic axis. Asymmetric catalysis and materials science applications are being investigated.^146^

#### 2.7.2. Synthesis of axillary chiral *N*-aryl benzimidazoles

There are many biological active compounds that include *N*-aryl benzimidazoles for instance, they are used as inhibitors of certain enzymes like nonpeptide thrombin.^147,148^ They are also important components of high-temperature polymers, veterinary medications, fungicides, herbicides, and dyes. Previously reported, dyes containing benzimidazole as structural component are utilized to protein fibers like wool and silk fabric, having properties including color features, color appearance in daytime and UV light, antibacterial activity, insect repellency, and to enhance wet adhesion properties.^149–151^ Axially chiral compounds play crucial structural roles in biologically active chemicals and natural products. Additionally, chiral ligand and catalytic components are preferred. Significant progress has recently been made in the asymmetric method for synthesizing axially chiral skeletons. To the greatest extent of our understanding, the atroposelective production of *N*-aryl benzimidazoles has been a very challenging process up to this point. A crucial organocatalyst in the asymmetric synthesis is the axially chiral phosphoric acids (CPAs).^152–154^ Researcher developed an organocatalytic, atroposelective process for making axially chiral *N*-aryl benzimidazoles that involved the breakage of carbon (C–C) bonds in the presence of CPAs. The CPAs showed a number of distinct advantages, including environmental friendliness, ease in cleaving the carbon–carbon bonds of multicarbonyl substances, excellent efficiency along with enantioselectivity, a wide range of substrate tolerance, as well as gram-scale chemical reactions without departing reactivity and enantioselectivity (ee).

**Scheme 17:**
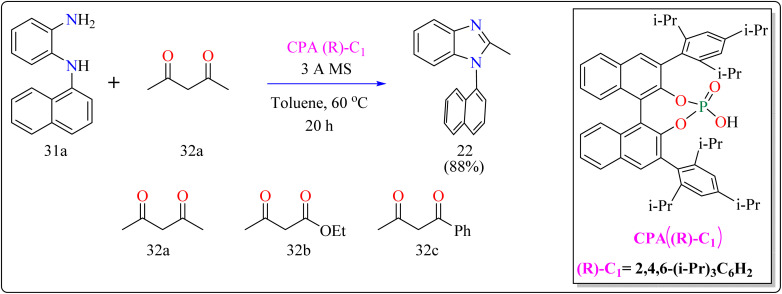
Optimized reaction conditions for CPAs catalyzed synthesis of auxiliary *N*-Aryl benzimidazoles.

**Scheme 18:**
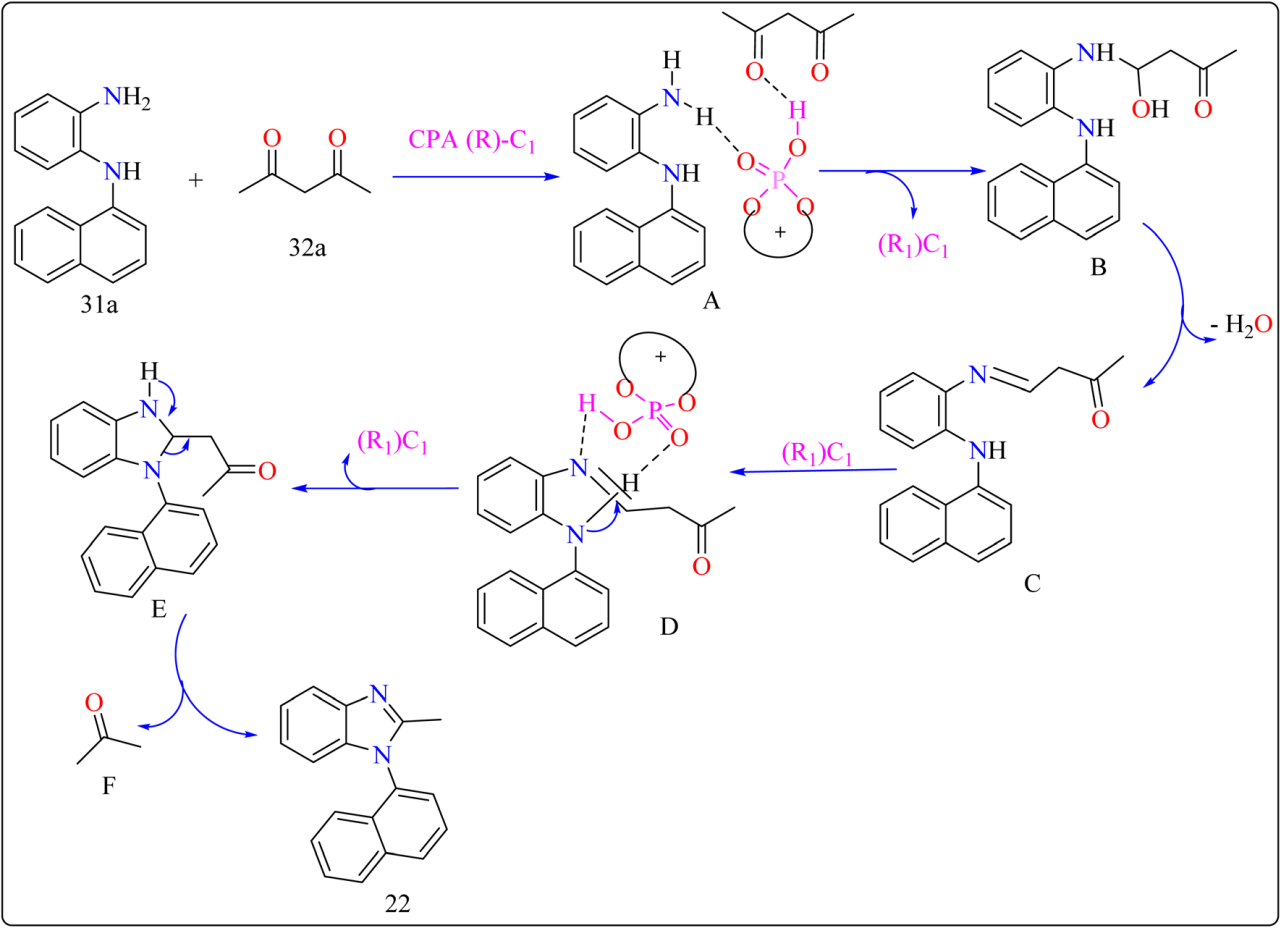
Proposed reaction mechanism for CPA catalyzed compound 22.

For the synthesis of compound 22, combined *N*^1^-(naphthalen-1-yl)benzene-1,2-diamine (31a) along with acetylacetone (32a) with addition of 10 mol% of CPA ((R)-C1), magnesium sulphate (MgSO_4_) in toluene, two additives sodium sulphate (Na_2_SO_4_) and 3 Å molecular sieve (MS), have been used for higher enantioselectivity at 60 °C for 20 h and three angstrom MS produced 88% yield and 92% enantioselectivity (Scheme 17). It was showed that 31a reacted with three-dicarbonyl molecules: 32a, ethyl acetoacetate (32b), and 1-phenylbutane-1,3-dione (32c). 32a and 32c exhibited a greater degree of reactivity (78–88% yields) as well as excellent ee (91 or 92%). According to Scheme 18, intermediate A was generated when two substrates, 31a and 32a, were treated with chiral phosphoric acid (R)-C_1_. Then, B is produced by the nucleophilic attack of the amino in 31a on the carbonyl in 32a, regenerating (R)-C_1_, and B was dehydrated to produce intermediate C. An intramolecular nucleophilic strike of the imino group on the atom of carbon found in the imine in D causes the catalyst to be released after the catalyst and C have reacted to form intermediate D. The target product, 22, was produced when F is separated from E by cleaving the C–C bond.^155^

### 2.8. Tetrahydrocarbazole catalyzed synthesis of biaryl and aryl-heteroaryls

Agrochemicals, fragrance, pharmaceuticals, and dyes are just a few of the many industries that use biaryl and aryl-heteroaryl complexes as important structural components.^156,157^ Biaryl or heteroaryl azo dyes are extensively researched for their potential uses in the textile industry, various clinical and biological studies, photochromic materials, indicators, sensors, and photo-sensitizers, as well as for their anti-microbial, antiseptic, and antioxidant properties.^158^ Furthermore, researcher discussed the preparation of biaryl and aryl-heteroaryl compounds utilizing an electron donor acceptor (EDA) complex^159^ produced by the reaction between the oxidant diazonium salt (33a) and the reductant tetrahydrocarbazole (THC), which has a high electron density. In particular, it was found that at milder conditions, EDA complex formed in catalytic levels was functional and facilitated a single electron transfer procedure without any photoactivation.

**Scheme 19:**
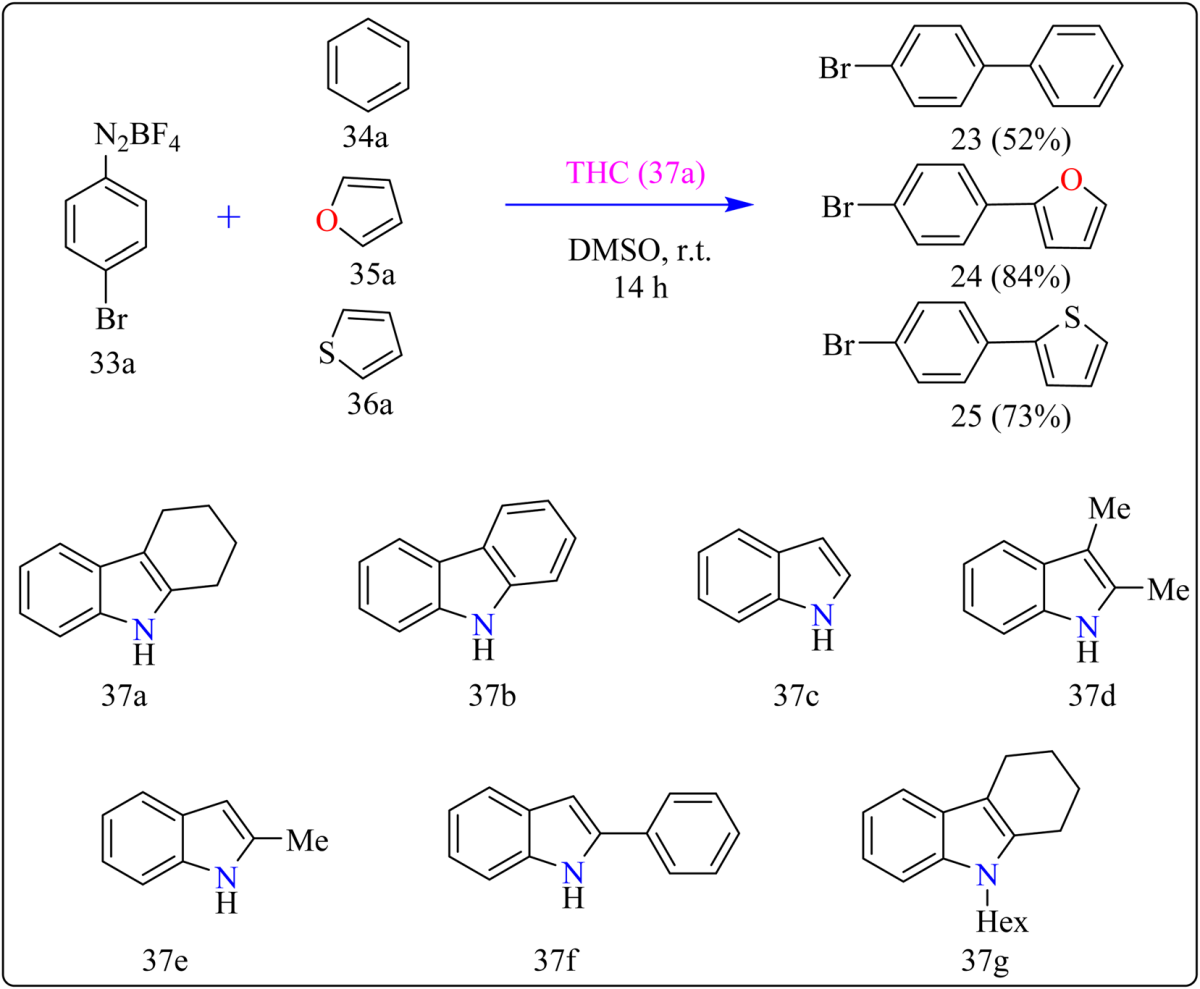
Optimize reaction conditions for the synthesis of biaryl and aryl-heteroaryls.

**Scheme 20:**
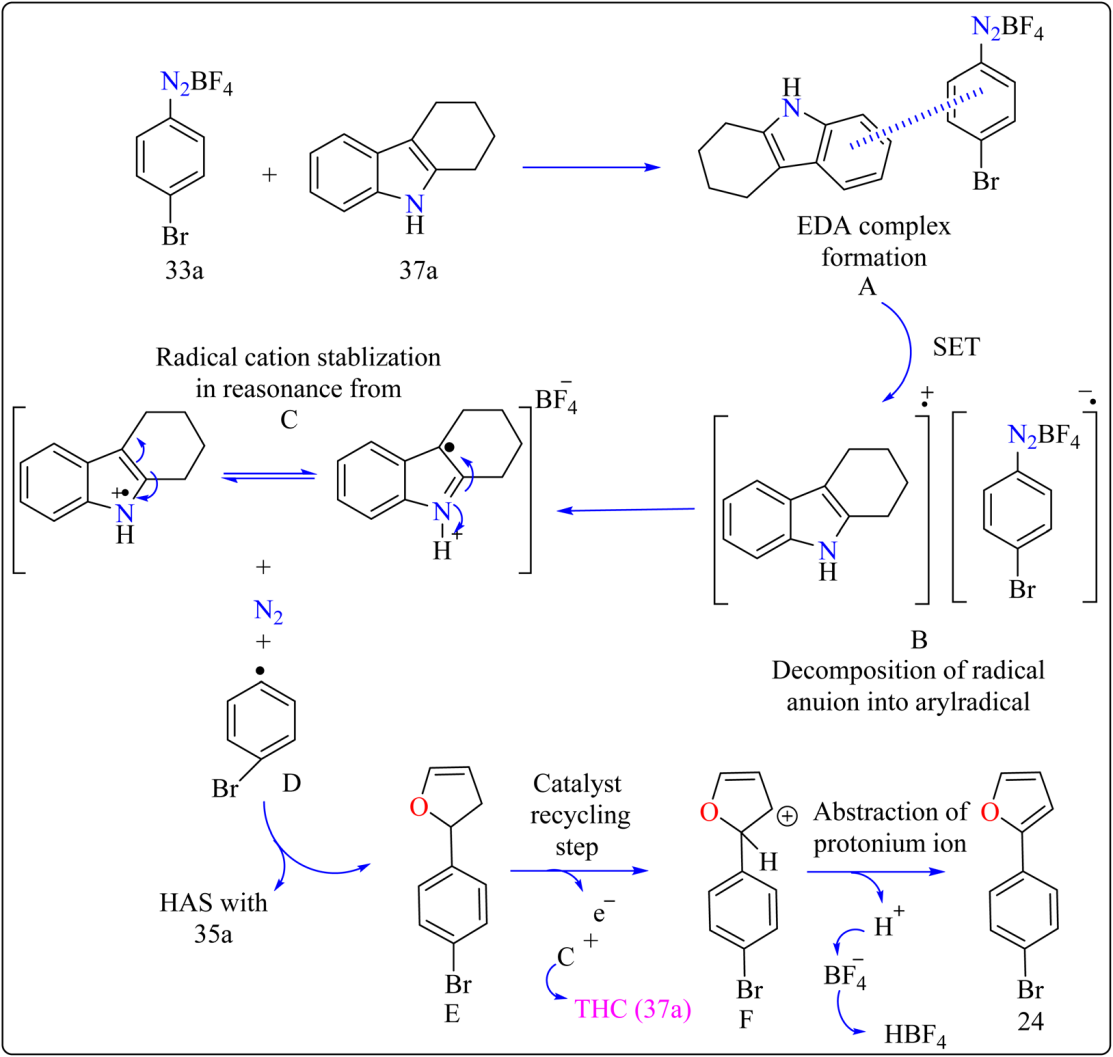
THC complex catalyzed reaction mechanism for compound 24.

For synthesis, Biaryl used one equivalent of 33a, Benzene (34a), and 10 mol% THC (37a), all of which were combined in a dark environment with N_2_ at the ambient temperature for 14 h. Similarly for synthesis of aryl-heteroaryl repeated same procedure with 20 equiv. of heteroarene (thiophene (35a), furan (36a)). It was shown that THC (37a) surpassed other catalysts with comparable structural characteristics like simple 37b, 37c, 37d, 37e, 37f, and 37g during catalytic screening (Scheme 19). DMSO proved to be the most effective of the various solvents tested. The 10 mol% of 37a catalyst provided maximum yield, conversely the yield decreased with 5 mol% of 37a but the 20 mol% had no apparent effect on the final yield. As shown in Scheme 20, the creation of the EDA complex (A) between 33a and 37a initiates the process. Without any photoexcitation, the created complex A was passed through an easy Single Electron Transfer (SET). The 37a, an organic reductant that contains nitrogen and is one of the elements that make up EDA, probably giving an electron to the component's 33a. Then generated radical cation B, radical anion species, and radical adduct likely breakdown into radical cation C, arylradical D, and nitrogen gas. The radical cationic entity C could be easily stabilized due to the existence of resonance patterns that include the stable tertiary free radical. Following a Hemolytic Aromatic Substitution (HAS) reaction with 35a, the arylradical would produce the radical species E. Currently, the intermediate C has the ability to remove one electron from the radical arylated species E and revert to its original catalytic form, the 37a molecule. After the borontetrafluoride anion deprotonated the cationic species F, the arylated compound 24 and HBF_4_ were produced as byproducts.^160^

### 2.9. Salicylic acid catalyzed reactions

Salicylic acid is a phenolic analogs, which are found in many plants, are essential for food preservation, cosmetics, medicines, and plant immunity.^161,162^ They play an important role as ligands in the catalysis of metal complexes in chemistry.^163^ Additionally, their application breadth in the fields of medicine and material science is broadened by the use of organocatalysis in a variety of synthetic transformations, including arylation, hydrodeamination, oxidative acylation, along with the synthesis of different biologically active derivatives.^164–166^

#### 2.9.1. Synthesis of α-sulfonyl ketoximes

α-Sulfonyl ketoximes are useful in chemical synthesis and biologically active due to their variety of functional groups. They function as adaptable building blocks with a wide range of synthetic possibilities for different organic chemistry applications.^167,168^ A number of synthesis techniques have been described for α-sulfonyl ketoximes, mainly based on radical-mediated oximation and arylsulfonylation.^169,170^ Few organocatalytic techniques are known, and metal-catalyzed procedures predominate. Salicylic acid has been shown to have the ability to act as an organocatalyst in the synthesis of aryl radicals from arylamines and *t*-butyl nitrite, providing a new route for this reaction. Furthermore, DABSO improves ease and safety in these reactions by acting as a secure replacement for sulfur dioxide.^171^ These biologically active molecules require more investigation into organocatalytic production from easily accessible starting materials.

**Scheme 21:**
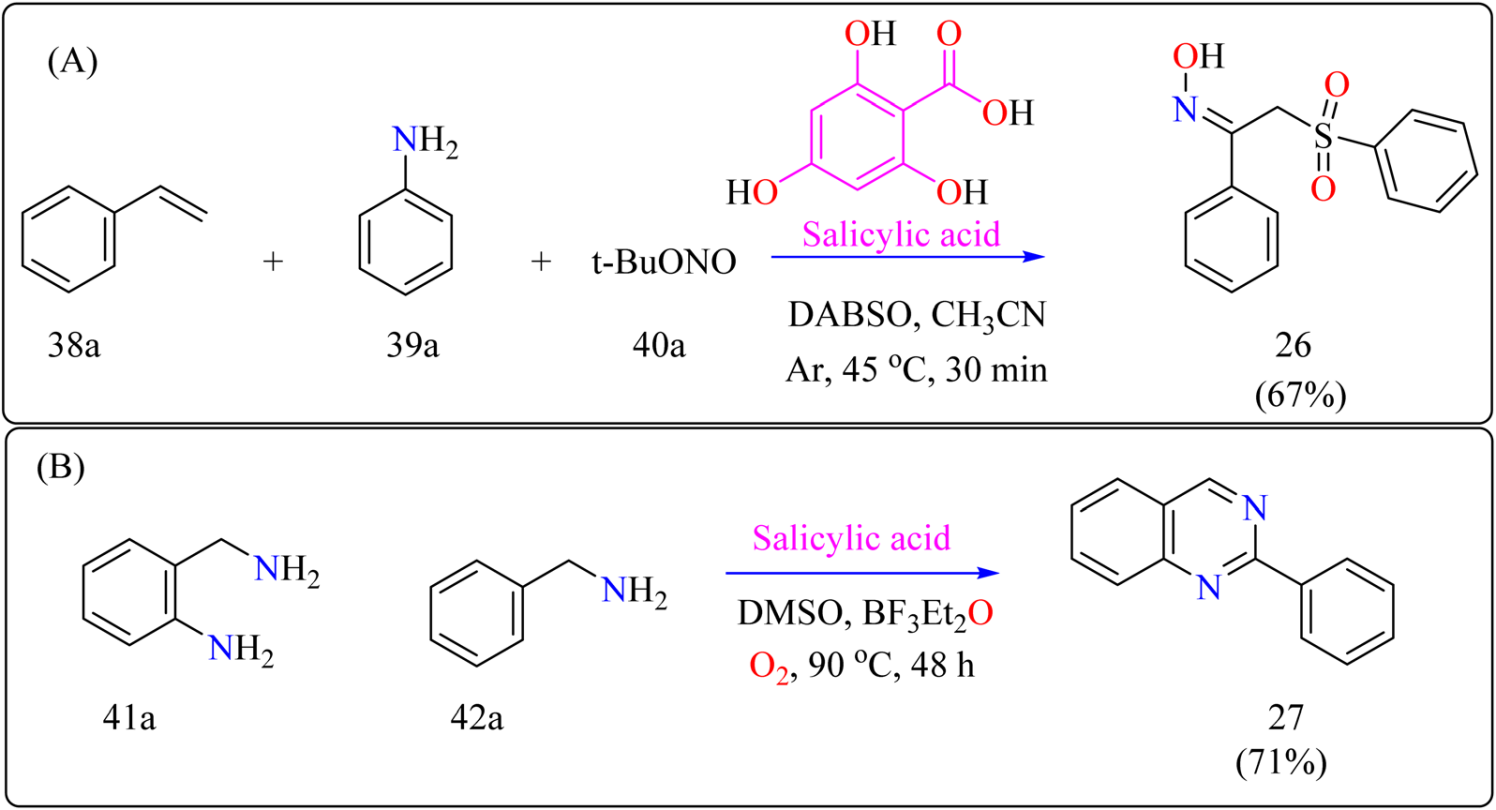
At optimized reaction conditions methodology for the synthesis of (A) α-sulfonyl ketoximes (26) and, (B) quinazolines (27).

The first organocatalytic synthesis of α-sulfonyl ketoximes was reported by the researchers (Scheme 21A). This was achieved using a four-component reaction including styrene derivative (38a), anilines (39a), *t*-butyl nitrite (40a), and DABSO, all of which were catalyzed by salicylic acid.^172^ Salicylic acid (10 mol%) aided in the production of product 10a in 51% yield in acetonitrile under argon at the ambient temperature in 30 minutes, supporting its catalytic role. Maintaining 1.5 equiv. of DABSO and 3.0 equiv. 40a, optimization yielded a 67% yield at 45 °C. Optimal conditions were demonstrated by additional modifications to the reaction temperature and reactant loadings. Other examined solvents fared worse than acetonitrile. These results define effective parameters for this novel organocatalytic synthesis.^173^

#### 2.9.2. Synthesis of quinazolines

Modern developments in materials science and medicine require more environmentally friendly synthetic processes and fundamental chemicals with greater purity.^174–176^ Using oxygen and salicylic acid, a metal-free oxidation catalyst method allows the synthesis of a variety of functional compounds from unstable imines in a single pot.^177^ This approach achieves great selectivity and environmental sustainability, making it suitable for multistep reactions such as the synthesis of quinazolines.^178^ For industrial and medicinal chemistry, the development of scalable, metal-free synthesis techniques for quinazolines is essential, highlighting the need for better environmental conditions and wider use.^179^ For synthesizing compound 27 as shown in Scheme 21B, salicylic acid (5 mol%), BF_3_–Et_2_O (10 mol%) as an additive, 2-aminobenzylamine (41a), benzylamine (42a), and DMSO as a solvent (1.0 mL) were all mixed in a 10 mL two-necked flask with an O_2_ atmosphere at 25 °C as part of a reaction setup. In an O_2_ environment, the mixture was agitated for 48 hours at 90 °C. Using the activated alumina as the filler and iso-hexane for elution, column chromatography was used for purification following the reaction to produce product 27.^180^

#### 2.9.3. Synthesis of *N*-heteroaryl stilbenoids

Stilbenoids are the structural component of squaraine dyes that are widely used in solar cell pigments, materials for non-linear optics, two-photon absorption compounds, NIR emitting fluorescent dyes, vivid violet, blue, or blue-green dyes came to be of enormous significance, components for biosensors and fluorescence imaging materials.^181^ For the production of medications, imaging agents, and materials such as organic light-emitting diodes, methyl *N*-heteroarenes must be olefinated.^182–184^ Aldehyde condensation or multi-step reactions are used in traditional procedures.^185–187^ Recent methods include using secondary or benzyl amines for metal-free deaminative olefination; however, more widespread use of environmentally friendly and effective catalytic systems free of metal residues is still desired.^188–190^

**Scheme 22:**
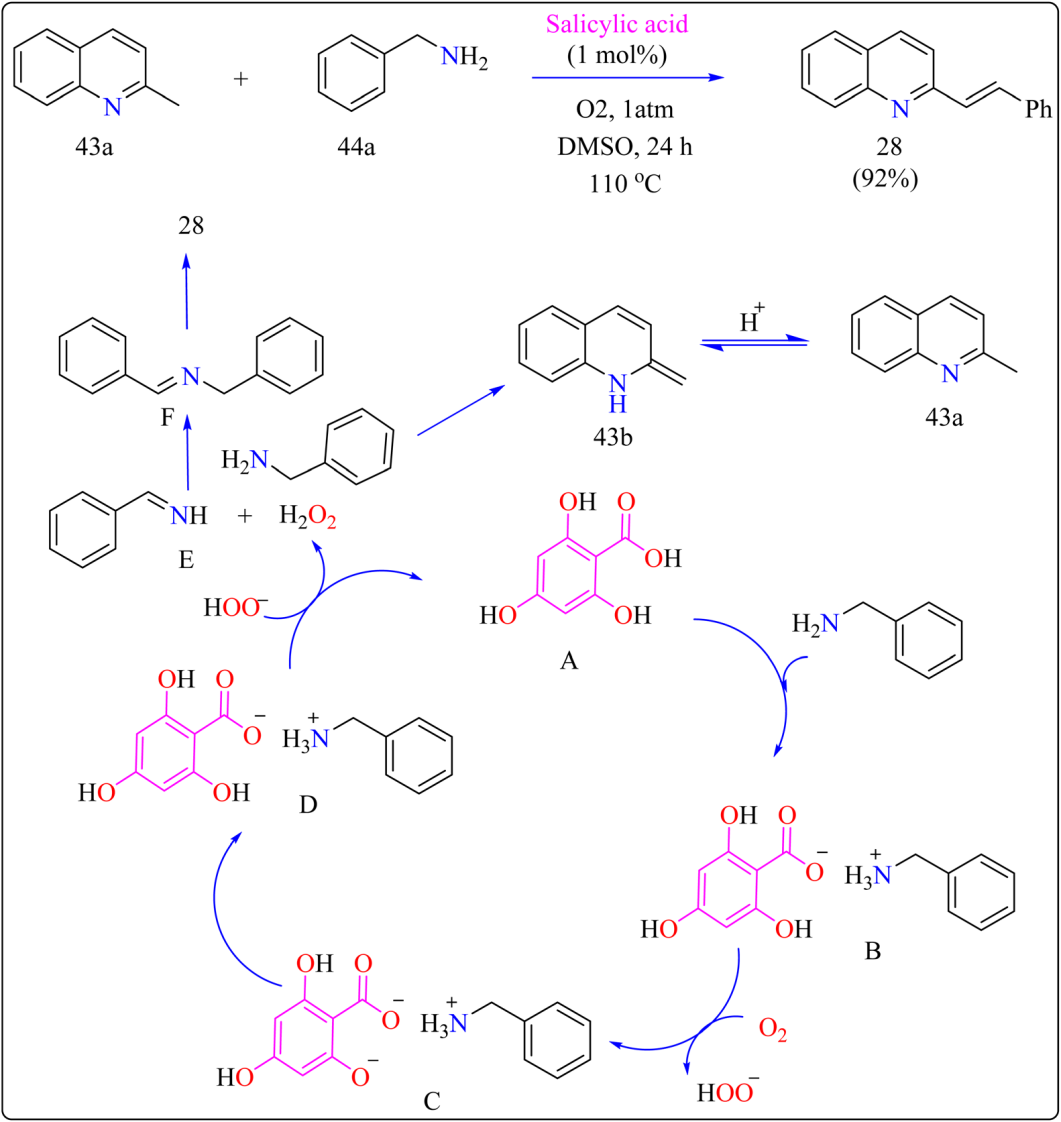
Schematic diagram for Gram-scale preparation of (*E*)-2-styrylquinoline.

Scheme 22 showed, 2-methylquinoline (43a), benzyl amine (44b), 4,6-dihydroxysalicylic acid (A), and DMSO (4 mL) were added to a 10 mL Schlenk tube and agitated at room temperature in the presence of an O_2_ balloon. Next, a warmed oil bath at 110 °C was used to hold the reaction mixture for 24 h. Further, the mixture was cooled with 5 mL of H_2_O and extracted with ethyl acetate. Drying over sodium sulphate and subsequent filtering and concentration of the mixed organic layer. Compound 28 was obtained by purifying the residue using column chromatography on silica gel in which petroleum ether/ethyl acetate served as an eluent. Optimized reaction experiments concluded that, 92% of the 28 yield was obtained by reducing the salicylic acid catalyst to 1 mol%. The reaction was hampered by lower temperature and shorter times. Yield was somewhat impacted by extending the reaction time to 30 h. Utilizing solvents such as DMF, toluene, and CH_3_CN, as well as reducing 44a equiv., decreased yield. An excess of DMSO and a lack of O_2_ reduced output.^191,192^

### 2.10. DBU catalyzed synthesis of quinolines

In organic synthesis, 1,8-diazabicyclo[5.4.0]undec-7-ene (DBU) an inexpensive, easily accessible organic base with a p*K*_a_ of 12 and due to low nucleophilicity is frequently utilized for multicomponent reactions, cyclizations, and chemical derivatizations.^193,194^ It is useful in various reactions due to its steric hindrance, stability, and ease of handling. Additionally recoverable, DBU finds application as a complexing ligand, catalyst, and base in a variety of practical applications.^195^

**Scheme 23:**
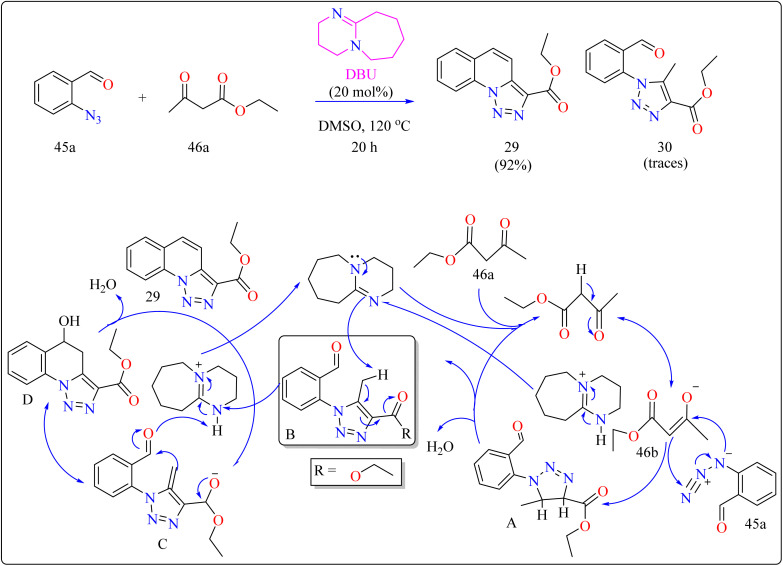
Methodology and reaction route for the synthesis of DBU catalyzed compound 29.

Quinolines are a type of heterocycles that exist both naturally and artificially and have fascinating biological properties.^196^ Because of their many pharmacological properties, including anti-malarial, anti-oxidants, anxiolytic, and antitumoral effects, compounds with quinolinic cores in their structures have garnered a lot of attention in the field of material science and has the potential to act as an antileishmanial agent.^197–200^ In previous years, researcher reported triazolo quinoline-based synthesis of complexes catalyzed by copper iodide, copper(ii), palladium catalyst and oxidative catalyst-free N–N coupling reaction was used to synthesize triazole derivatives.^201–204^ The creation of more effective catalytic methods is still a major issue in synthetic organic chemistry, despite the notable progress made towards the preparation of quinoline-triazole hybrids and catalyzed by metal free DBU organocatalyst. This is a straightforward and effective method involving 2-azidobenzaldehyde (45a) and carbonyl compounds (46a) and DBU (20 mol%) as a catalyst was used for the organocatalytic synthesis of several fused quinolines 29 and traces of compound 30, shown in Scheme 23. For synthesizing compound 29, the enolate 46b was formed by DBU eliminating one methylene acidic proton from molecule 46a. Then, a [3 + 2] cycloaddition event occurs between enolate 46b and 45a, producing the intermediate triazoline A, which dehydrates to generate the intermediate B. The intermediate B then reacts with a catalyst, which extracts a proton from the methyl group at the 5-position of triazole B, with electron displacement by the triazole nucleus and the carbonyl, generating the enolate intermediate C. The negative charge in the oxygen then migrates, causing the carbon linked at position 5 of the triazole to undertake a nucleophilic attack on the pendent *ortho*-carbonyl group, resulting in the intermediate D, which after dehydration formed the compound 29.^205^

## 3 Conclusion

As evident by the examples included in this paper, organocatalysis has already attained an advanced state of sophistication. The use of multidisciplinary research will undoubtedly result in the growth of this field of catalysis, in which organocatalyzed synthesis of various compounds. Because of the adaptability of organic synthesis and generally accepted theories on the effect of substituents on the electronic characteristics of organic compounds, organic components allow a significant degree of design flexibility with tunable qualities that are typically not feasible in inorganic materials. The idea can be supported theoretically by the growing number of synthetic compounds, and in the coming years, we might observe wider improvements in this field. This review's goal is to provide materials scientists, organic and industrial chemists, and other researchers with an overview of these developing advanced organocatalysts for simple eco-friendly organic transformations or building blocks of organic complexes. The basic idea of organo-catalysis originated from the use of simple and common organic molecules, readily available in the laboratory, to be used as catalysts for organic and inorganic transformations. It is highly recommended to the chemists to adopt this advanced idea and technology for chemical synthesis.

## Data availability

No primary research results, software or code have been included and no new data were generated or analysed as part of this review.

## Author contributions

A. Z.: writing – original draft, investigation. G. I.: review and editing. U. S. S.: data collection. F. J.: data curation and visualization. M. S.: formal analysis and validation. M. Y.: validation and review. Z. U.: data collection and formatting. M. A.: review and formatting. M. A. I.: conceptualization, resources, supervision, and overall guidance.

## Conflicts of interest

There are no conflicts to declare.
